# Immunoinformatics Study: Multi-Epitope Based Vaccine Design from SARS-CoV-2 Spike Glycoprotein

**DOI:** 10.3390/vaccines11020399

**Published:** 2023-02-09

**Authors:** Ramadhita Umitaibatin, Azza Hanif Harisna, Muhammad Miftah Jauhar, Putri Hawa Syaifie, Adzani Gaisani Arda, Dwi Wahyu Nugroho, Donny Ramadhan, Etik Mardliyati, Wervyan Shalannanda, Isa Anshori

**Affiliations:** 1 Lab-on-Chip Group, Department of Biomedical Engineering, School of Electrical Engineering and Informatics, Bandung Institute of Technology, Bandung 40132, Indonesia; 2 Nano Center Indonesia, Jl. Raya Puspiptek, South Tangerang 15314, Indonesia; 3 Research Center for Pharmaceutical Ingredients and Traditional Medicine, National Research and Innovation Agency (BRIN), Cibinong 16911, Indonesia; 4 Research Center for Vaccine and Drug, National Research and Innovation Agency (BRIN), Cibinong 16911, Indonesia; 5 Department of Telecommunication Engineering, School of Electrical Engineering and Informatics, Bandung Institute of Technology, Bandung 40132, Indonesia

**Keywords:** antigenic epitope, epitope prediction, spike glycoprotein, TLR-3, LR-4

## Abstract

The coronavirus disease 2019 outbreak has become a huge challenge in the human sector for the past two years. The coronavirus is capable of mutating at a higher rate than other viruses. Thus, an approach for creating an effective vaccine is still needed to induce antibodies against multiple variants with lower side effects. Currently, there is a lack of research on designing a multiepitope of the COVID-19 spike protein for the Indonesian population with comprehensive immunoinformatic analysis. Therefore, this study aimed to design a multiepitope-based vaccine for the Indonesian population using an immunoinformatic approach. This study was conducted using the SARS-CoV-2 spike glycoprotein sequences from Indonesia that were retrieved from the GISAID database. Three SARS-CoV-2 sequences, with IDs of EIJK-61453, UGM0002, and B.1.1.7 were selected. The CD8+ cytotoxic T-cell lymphocyte (CTL) epitope, CD4+ helper T lymphocyte (HTL) epitope, B-cell epitope, and IFN-γ production were predicted. After modeling the vaccines, molecular docking, molecular dynamics, in silico immune simulations, and plasmid vector design were performed. The designed vaccine is antigenic, non-allergenic, non-toxic, capable of inducing IFN-γ with a population reach of 86.29% in Indonesia, and has good stability during molecular dynamics and immune simulation. Hence, this vaccine model is recommended to be investigated for further study.

## 1. Introduction

The COVID-19 pandemic, caused by the SARS-CoV-2 virus, has become a world-wide issue. SARS-CoV-2 is a beta coronavirus in the Coronaviridae family and is believed to have originated from bats. It is most closely related to BatCoV-RaTG13, with a sequence similarity rate of approximately 96%, and to SARS-CoV, with a similarity rate of roughly 80% [1,2]. The SARS-CoV-2 virus has a positive, and single-stranded RNA with 29.8 kilobases (kb) long. This genome encodes non-structural proteins (Open Reading Frames (ORFs) a and b) and structural proteins (Envelope (E), Spike (S), Nucleocapsid (N), and Membrane (M)) [3,4]. The spike glycoprotein of SARS-CoV-2 is comprised of two subunits: S1 (residues 14 to 685) and S2 (residues 686 to 1273) and has a total length of 1273 amino acids (AA). The S1 subunit contains N-terminal and receptor-binding domains (RBD), which play a critical role in the infection process as it binds to the ACE2 receptor in human cells [5,6]. Research has revealed the potential of the spike glycoprotein as an antigenic region [7]. This discovery makes it a promising focus for research and vaccine development.

Vaccines trigger the production of specific antibodies against a disease, but the development of conventional vaccines made from the whole virus can lead to side effects [8], and potential reverse virulence [9,10]. The presence of viral mutations can also change the infection mechanism so that a new vaccine is needed to induce the specific antibodies [9,11]. In Indonesia, there are several types of COVID-19 vaccines available, including mRNA-based vaccines (Pfizer and Moderna), adenovirus vector-based (AstraZeneca), and inactivated virus vaccine (Sinovac). The main challenge in the development of the COVID-19 vaccine is the high mutation rate and transmissibility of the virus [12]. Thus, a platform that can boost vaccine design in a shorter time is crucial to minimize the adverse effect on public health conditions. To address this, immunoinformatics is being used to design vaccines more efficiently, providing a precise, rapid, and effective approach to vaccine development [13]. Utilizing large immunoinformatics databases can support multiepitope vaccine design, which has been shown to induce an immune response against a specific virus without side effects [14]. The use of peptide series in multiepitope vaccines can prevent and treat viral infections by inducing an immune response from CTL, Th, and B cells [15]. Adding a linker adjuvant between each epitope can also enhance the vaccine’s immunogenic effect [10]. Therefore, an immunoinformatic study to see the potential of a multiepitope vaccine for SARS-CoV-2 can help to foresee alternatives in developing vaccines for the global population.

In this study, we performed an immunoinformatic analysis to design a multiepitope vaccine for three SARS-CoV-2 S proteins detected in Indonesia. A previous study by Gustiananda et al. (2021) [16] revealed polyprotein sequences for vaccine candidates based on the COVID-19 ORF1ab. They were validated using the physicochemical structure and analyzed further through cross-reactivity, molecular docking, and immune simulation. Meanwhile, Febrianti and Narulita (2022) [17] designed a multiepitope COVID-19 vaccine using spike glycoprotein and validated its sequence using physicochemical structure, which also continued to the design of a plasmid vector. Regarding these studies, our research started by constructing a phylogenetic tree from the evolution of the target sequence in Indonesia, searching the epitope of T and B cells, constructing and validating the vaccines and their 3D structure, docking them with TLR-3 or TLR-4, performing a molecular dynamics study, in silico immune simulations, and constructing a plasmid vector.

## 2. Materials and Methods

### 2.1. Study Design

**Figure 1:**
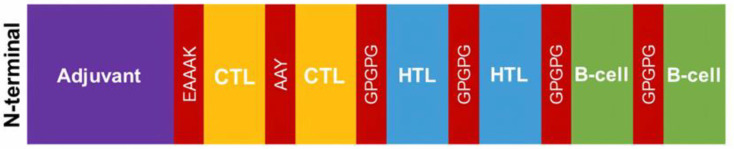
Epitope grouping sequence on the vaccine structure and its linkers.

The study started by retrieving Indonesia’s sequence of SARS-CoV-2 spike glycoprotein from the database. These data were converted into amino acid sequences, then phylogenetic tree was constructed Three sequences were selected for further analysis against the original sequence (Wuhan-Hu-1). These sequences were used to predict CTL epitopes, HTL epitopes, B-cell epitopes, and IFN-γ. The antigenicity, allergenicity, toxicity, and population coverage were analyzed. A multiepitope vaccine was then designed, modeled, and validated. Finally, molecular docking, molecular dynamics analysis, and in silico immune simulations were performed. Figure 1 shows the design of this study.

### 2.2. The Sequences of SARS-CoV-2 Spike Glycoprotein in Indonesia

The nucleotide sequences of SARS-CoV-2 were retrieved from the Global Initiative on Sharing Avian Influenza Database (GISAID) (https://gisaid.org/, accessed on 13 January 2021) [18], on 19 January 2021. The sequences obtained were the viral sequences that appeared in Indonesia. Meanwhile, the nucleotide sequences of SARS-CoV-2 Wuhan-Hu-1 were retrieved from the National Centre for Biotechnology Information (NCBI) (https://www.ncbi.nlm.nih.gov/, accessed on 16 January 2021) (Accession ID: NC_045512). During this research, a new variant virus occurred in Indonesia in March 2021, the SARS-CoV-2 B.1.1.7. Its sequence was retrieved from GISAID and included in the analysis. All the nucleotide sequences retrieved were converted into amino acids using the MEGA-X software package [19]. Then, multiple sequence alignment was performed using ClustalW [20]. The phylogenetic tree was constructed with maximum likelihood and the neighbor-joining methods by MEGA-X [19,20,21,22]. The sequence at the outer clade of the phylogenetic tree was chosen to be further analyzed as it represents the most common sequence that spread in Indonesia (ID EPI_ISL_574613). Meanwhile, the sequences with the furthest clade were also selected as the most distinct sequence and showed the lowest similarity (ID EPI_ISL_576116).

### 2.3. CTL Epitope Prediction

The CTL epitopes were identified through NetCTL 1.2 web server (https://services.healthtech.dtu.dk/service.php?NetCTL-1.2, accessed on 2 February 2021) [23], using A1, A2, A3, A24, A26, B7, B8, B27, B39, B44, B58, and B62 as the supertypes. The selected epitope threshold was 0.95 with a specificity of 0.98, then validated for compatibility with the HLA allele of the Indonesian population (Table 1) using the NetMHC4.0 web server (https://services.healthtech.dtu.dk/service.php?NetMHCpan-4.0, accessed on 10 February 2021) [24]. The HLA alleles were collected using classical methods from The Allele Frequency Net Database (AFND) and the gold standard population [25]. Epitopes were selected based on the predicted IC_50_ score <10 μM and 0.06 percentile [26,27,28,29].

### 2.4. HTL Epitope Prediction

The HTL epitopes were identified using NetMHCIIpan4.0 (EL + BA) (https://services.healthtech.dtu.dk/services/NetMHCIIpan-4.0/1-Submission.php, accessed on 13 February 2021) [30]. The threshold used was 0.1 and IC_50_ < 10 μM. The HLA alleles of the Indonesian population were used as listed in Table 1.

### 2.5. B-Cell Epitope

Linear B cell epitopes were identified using the Bepipred2.0 web server (http://www.cbs.dtu.dk/services/BepiPred/, accessed on 15 February 2021) [31]. Meanwhile, the conformational prediction of B-cell epitopes was predicted using the ElliPro web server (http://tools.iedb.org/ellipro/, accessed on 17 February 2021) [32]. The selected Ellipro prediction was set by scoring >0.5 with 5–20 AA length.

### 2.6. IFN-γ Prediction

The IFN-γ stimulation test was performed using IFN-epitopes (https://webs.iiitd.edu.in/raghava/ifnepitope/, accessed on 28 February 2021) for selected HTL and B-cell epitopes. The parameters used were no-splitting at window length, the motif and SVM hybrid algorithm, also the prediction model of IFN-γ versus non-IFN-γ.

### 2.7. Antigenicity, Allergenicity, Toxicity, and Population Coverage Analysis

The selected epitopes were analyzed for antigenicity using Vaxijen 2.0 (http://www.ddg-pharmfac.net/vaxijen/VaxiJen/VaxiJen.html, accessed on 17 March 2021) with a threshold of 0.5 [33]. The allergenicity testing was carried out using AllerTopv2.0 (https://www.ddg-pharmfac.net/AllerTOP/method.html, accessed on 20 March 2021) [34] and the toxicity testing was conducted using ToxinPred (https://webs.iiitd.edu.in/raghava/toxinpred/index.html, accessed on 23 March 2021) [35]. The expected vaccine coverage for the Indonesian population was predicted using the IEDB population coverage (http://tools.iedb.org/population/, accessed on 31 March 2021) [36].

### 2.8. Multiepitope Vaccine Construction, Modeling, and Validation

The selected epitopes were utilized to design the multiepitope vaccine. Beta-defensin (NCBI Accession ID: P81534) was used as an adjuvant at the N-terminal [37]. The linker EAAAK was used to link the adjuvant to the CTL, and the linker AAY was used to link the CTL–CTL epitopes. The linker GPGPG was used to connect CTL and HTL epitopes, among HTL epitopes, HTL and B cell epitopes, and among B cell epitopes (Figure 1). The 3D structure of the predicted vaccines was built using TrRosetta (https://yanglab.nankai.edu.cn/trRosetta/, accessed on 17 April 2021) [38]. Then, the structural accuracy of the vaccine model was evaluated using PROCHECK (http://www.ebi.ac.uk/thornton-srv/software/PROCHECK/, accessed on 22 April 2021) [39,40,41]. The structural quality of the vaccine model was measured using ERRAT (https://saves.mbi.ucla.edu/, accessed on 23 April 2021) with a threshold > 50 (maximum value 100) [42].

### 2.9. Molecular Docking

The vaccine models were docked against TLR-3 and TLR-4 (PDB ID: 7C76 and 3FXI) using the HADDOCK2.4 web server (https://wenmr.science.uu.nl/haddock2.4/, accessed on 1 May 2021) [43,44]. The binding sites selected were all epitopes and adjuvants (without linkers). The binding sites for TLR-3 were ASN541, HIS539, GLY360, GLY361, SER362, THR363, LEU364, GLU368, GLU557, THR559, SER562, GLU564, GLU565, and SER566 [45,46] while for TLR-4 were ARG264, ASP294, TYR296, LYS341, LYS362, SER416, ASN417, GLY439, and GLN436 [47]. The binding affinity was measured using the PRODIGY web server (https://wenmr.science.uu.nl/prodigy/, accessed on 3 May 2021) with a temperature setting of 25 °C [48,49]. The types and distances of interactions between the vaccine model and TLR-3 or TLR-4 were then visualized using the PDBSum Generate web server (http://www.ebi.ac.uk/thornton-srv/databases/pdbsum/Generate.html, accessed on 4 May 2021) [50,51].

### 2.10. Molecular Dynamics

Molecular dynamics simulation was performed using GROMACS and CHARMM36 force field for each vaccine model and its complex against TLR3 or TLR4. The simulation was performed using TIP3P as a water model and with 0.15 NaCl by adding the appropriate number of ions (sodium or chloride). Periodic boundary conditions (PBCs) were applied to the system in all the spatial directions. LINCS algorithms were used, and all hydrogen bonds were constrained. A 1.2 nm distance cutoff for the short-distance electrostatic and Van der Waals interactions was used. Particle Mesh Ewald algorithm (PME) was used to calculate the long-range electrostatic forces. The steepest descent algorithm was used to minimize the system’s energy. The system was then allowed to reach an equilibrium state through the NVT ensemble by using the V-Rescale thermostat at 300 K, then through the NPT ensemble by using the Parrinello–Rahman barostat at 1 atm. A 50 ns simulation was performed for the individual vaccine model and 10 ns simulation was performed for the complex system.

### 2.11. In Silico Cloning and Immune Simulation

The designed vaccine was simulated using a C-ImmSim web server (https://kraken.iac.rm.cnr.it/C-IMMSIM/, accessed on 3 August 2021) [52,53]. The simulation parameters were kept default except for the time steps in 1, 84, and 170 with 1050 total simulation phases. Hence, there were three injections at the interval of four weeks [54]. In order to optimize the expression of the designed vaccine, codon optimization, and cloned vector were conducted. Codon optimization was done using the VectorBuilder (https://en.vectorbuilder.com/tool/codon-optimization.html, accessed on 7 August 2021). After optimization, the vaccine sequence was inserted into the pET28a-EgC plasmid vector from the Addgene (https://www.addgene.org/, accessed on 8 August 2021) with XbaI and XhoI restriction sites using Serial Cloner software.

## 3. Results and Discussion

### 3.1. Evolutionary Relationship of SARS-CoV-2 in Indonesia and Detection of Its Mutation Site

In this study, a phylogenetic tree was built to analyze the genetic changes of SARS-CoV-2 in Indonesia and understand how new virus variants may emerge. A total of 166 SARS-CoV-2 spike glycoprotein nucleotide sequences collected from GISAID and sampled from Indonesia were analyzed. The sequences shared a common ancestral lineage to the wild type of SARS-CoV-2, Wuhan-Hu-1. Based on the constructed phylogenetic tree (Table S1) the EIJK-61453 has the most similarity to the Wuhan-Hu-1, with 100% similarity, while the UGM0002 has the least similarity, with 99.84% similarity. These two sequences along with a later emerged sequence, B.1.1.7 were then used for further analysis. The mutation of SARS-CoV-2 has occurred over time and led to changes in its properties, this may affect the human antibodies’ recognition. Several studies have reported that any mutation in the SARS-CoV-2 spike protein can affect neutralization.

Compared to Wuhan-Hu-1, there were mutations in UGM0002 and B.1.1.7 except EIJK-61453. The mutated amino acids are listed in Table 2. The deletion of H69 and V70 results in two-fold higher infectivity than the Wuhan-Hu-1 and decreases the sensitivity of antibodies to neutralize the virus. The N501 mutation into Y increases the ability of the virus to bind to ACE2. The D614 change into G that occurred in UGM0002 and B.1.1.7 also causes an increase in the infectivity of the SARS-CoV-2 to neutralizing antibodies [55,56,57,58]. However, Korber’s study revealed that this does not imply that the virus is antibody-resistant [59]. In addition, the P681 mutation into R at the furin cleavage site has been shown to increase in the membrane fusion ability, leading to enhanced transmission of the virus in the body [60]. The impact of other mutations remains unknown.

The mutation on SARS-CoV-2 spike glycoprotein changes the segments targeted by antibodies. Several studies reported that these mutations can evade monoclonal antibodies [56,61] and influence polyclonal antibody recognition [62,63]. These mutations have been found to be present in the human population [61].

### 3.2. Identification and Selection of T-Cell Epitopes

A total of 300 identified CTL epitopes have been identified. The final list of the selected epitopes for each sequence is presented in Table 3. The epitopes were chosen based on low percentile and the IC_50_ value. Antigenicity, allergenicity, as well as toxicity were considered. As a result, seven epitopes from each Wuhan-Hu-1 and EIJK-61453, six epitopes from UGM0002, and five epitopes from B.1.1.7 were shortlisted for further multiple-epitope-based vaccine design.

The selected HTL epitopes are listed in Table 4. There were five selected HTL epitopes for each Wuhan-Hu-1 and EIJK-61453, four epitopes for the UGM0002, and three epitopes for the B.1.1.7. The selection of epitopes was also based on the value of IFN-γ, a cytokine mainly produced by Natural Killer (NK) cells. IFN-γ plays a vital role in inhibiting viral replication. The presence of antiviral IFN-γ in APC can elevate the stimulation of the adaptive immune response to the infection, resulting in the generation of memory in the body to recognize, and neutralize the virus if a similar infection occurs in the future. The presence of IFN-γ stimulation also increases the antigen presentation process during T-cell priming, both in terms of efficiency, quantity, quality, and diversity of peptides loaded into the MHC-I receptor [64,65]. Therefore, the epitope selected for vaccine construction is preferably derived from an epitope capable of stimulating IFN-γ.

### 3.3. Identification and Selection of B-Cell Epitopes

B-cell epitopes consist of linear and discontinuous (conformational) epitopes with 5–20 AA lengths. Its selections were based on an antigenicity score higher than 0.5 and the IFN-γ stimulation ability. Table 5 shows selected linear B-lymphocyte (LBL) epitopes that meet the criteria. There were five selected LBL epitopes from each Wuhan-Hu-1, EIJK-61453, and B.1.1.7, also six selected LBL epitopes from UGM0002. Table S4 shows two discontinuous B-cell epitopes shortlisted from each sequence.

### 3.4. Indonesian Population Coverage from Selected Epitopes

Each epitope can bind to a specific HLA allele depending on the population coverage. Therefore, in this study, the coverage of the Indonesian population was analyzed to determine the specific epitope that might bind to the Indonesian HLA allele. The selected epitopes of Wuhan-Hu-1 and EIJK-61453 have a coverage score of 78.26%, while the selected epitope of UGM0002 and B.1.1.7 have 86.29% and 84.28% coverage scores, respectively. Tables S1–S3 show at least one HLA from Austronesian Indonesian ethnicity can bind to the selected CTL and HTL epitopes. The details of the coverage distribution of each epitope are attached in Figure S2.

### 3.5. Epitope Analysis in Human Peptide

The mimicry of the epitope needs to be considered in the construction of a multi-epitope-based COVID-19 vaccine as it can result in an auto-immune effect [66]. Clinical reports suggest that no T-cell and B-cell epitopes for the SARS-CoV-2 vaccine candidate have been found to be homologous or share similarities with proteins that cause auto-immune effects or increase the severity of COVID-19 disease [67,68,69]. Another study found that there were epitopes with similarities to human peptides on six or seven consecutive peptides based on the results of CTL analysis using sequences from the IEDB database. KIYSKHTPI and SPRRARSVA are among those epitopes [70]. However, no reports specifically mention the effect of this peptide similarity, but this can be considered for further in vitro/in vivo analysis.

### 3.6. Vaccine Construction and Its Validation

The multi-epitope vaccine was constructed using all the selected epitopes by optimizing the arrangement of multiple epitopes connected by linkers and an adjuvant. The possible epitope combination was then evaluated by Ramachandran plot, TrRosetta, and ERRAT score. Three vaccines that fulfill the criteria are presented in Table S5. During the optimization, we found that the prediction score from TrRosetta can be optimized if the sequence is arranged in such a manner that is familiar to the existing protein sequence template, thus making the algorithm easier to recognize the vaccine structure. Moreover, AA length also affects the quality of the vaccine structure. The increasing number of AA will potentially reduce the structure’s quality in Psi–Phi stability and the TM-Score and ERRAT (Table S5).

**Figure 2:**
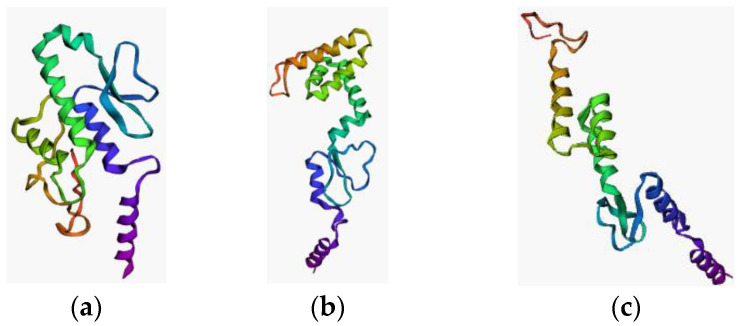
3D visualization of vaccine structure (**a**) EIJK-61453 (**b**) UGM0002 (**c**) B.1.1.7.

**Figure 3:**
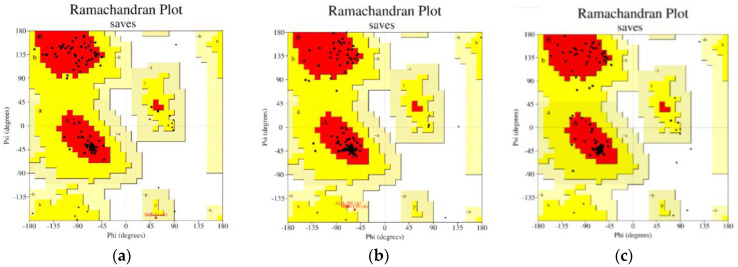
Ramachandran plot for (**a**) Wuhan-Hu-1 and EIJK-61453 Vaccine Model, (**b**) UGM0002 Vaccine Model (**c**) B.1.1.7 Vaccine Model.

Table 6 displays the optimal result of the vaccine construction prediction. Wuhan-Hu-1 and EIJK-61453 were found to be identical, only the EIJK sequence will be subjected to further analysis. The 3D visualization and Ramachandran plot of those are shown in Figure 2 and Figure 3, respectively. The use of linkers in multi-epitope vaccine development can primarily minimize the probability of deformation in the vaccine structure [71]. We utilized EAAAK, AAY, and GPGPG sequence linkers to build the complex vaccine and determine the best quality of the produced vaccine. The use of different linkers may also impart a specific role to the vaccine structure. For instance, the EAAAK linker avoids interference from other proteins when an adjuvant binds to its receptor. The other linkers such as AY and GPGPG were used to induce the immune response and provide a site for proteasomal cleavage. Furthermore, glycine and proline are often present in a stable beta turn. Proline’s cyclic structure is ideal for beta-turn, while glycine has the smallest side chain compared to all other AAs making it the most sterically flexible linker [72].

We also use an adjuvant to enhance the efficacy of our designed vaccine. In comparison to inactivated or attenuated virus-based vaccine, a peptide-based vaccine has lower immunogenicity, and therefore an adjuvant is needed. It can boost the immune response and acts as a vehicle to deliver the vaccine structure [10]. Moreover, different adjuvants can trigger varying immune responses. The Matrix-M™ adjuvant elicits TH1 immune response, while the AS03 adjuvant elicits both Th1 and Th2 cytokine responses [10]. In our study, Beta-defensin was used as an adjuvant and functioned as an immunomodulator and anti-microbial agent [73].

### 3.7. Docking Study of the Constructed Vaccine Model against TLR-3

**Figure 4:**
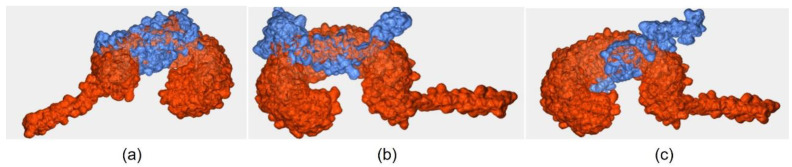
Docking of proteins between TLR-3 and (**a**) Wuhan-Hu-1 and EIJK-61453, (**b**) UGM0002 (**c**) B.1.1.7.

All three vaccine models were successfully docked with TLR-3, as shown in Figure 4. The details of the binding interaction are shown in Table 7. The binding affinity indicates the binding interaction of the two molecules. The docked complex of Wuhan-Hu-1-TLR-3 and EIJK-61453-TLR-3 present the exact same model with the binding affinity of −18.7 kcal/mol, and 0.5 ± 0.3 RMSD score. Meanwhile, the complex of UGM0002-TLR-3 and B.1.1.7-TLR-3 have binding affinities of −19.4 kcal/mol, and −15.6 kcal/mol.

TLR is a type of Pattern Recognition Receptor (PRRs) that functions as an early detector of pathogens. Signaling in the TLR will induce an innate and adaptive immune response against specific antigens. TLR-3 can recognize the presence of viral infection, therefore the existence of a strong complex between the vaccine model and TLR-3 is expected. Amino acid residues GLY360, GLY361, SER362, THR363, LEU364, GLU368, HIS539, ASN541, GLU557, THR559, SER562, GLU564, GLU565, and SER566 in TLR-3 can stimulate TLR-3 signaling. Among the residues, ASN541 facilitates the TLR-3 recognition to its target, while HIS539 plays a role in TLR-3 activation.

Among all the docked models, only the EIJK-61453 vaccine–TLR-3 complex has interactions with ASN541 (neutral), through PHE12 (aromatic) with non-bonded contacts. This interaction may occur between the CG atoms, OD1, and ND2, with an average distance of 3.57Å. Hydrogen bonds also occurred between TLR-3 HIS539 (positive) and LEU15 (aliphatic) with a distance of 2.70Å. The EIJK-61453 vaccine model also interacts with TLR-3 ILE566 at residues GLY137 and GLY138 in the form of nonbonded contacts. The interaction with residue ILE566 triggers the interaction of TICAM-1 and the transduction of IFN-β and NF-kB as an innate immune response. However, the docked complex UGM0002-TLR-3 and B.1.1.7-TLR-3 have no residues bound to TLR-3 HIS539 or ASN541.

The EIJK-61453-TLR-3 complex model has the most hydrogen bonds (18 hydrogen bonds) compared to the B.1.1.7-TLR-3 (11 hydrogen bonds) and UGM0002-TLR-3 model (12 hydrogen bonds). The B.1.1.7-TLR-3 model has four salt bridge interactions while the EIJK-61453-TLR-3 and UGM0002-TLR-3 have three salt bridge interactions. Intra-protein interactions in these complexes are different from the surface interactions between proteins. In intra-protein interactions, the increased number of hydrogen bonds contribute to the stability of the protein structure, while salt bridges can enhance the folding stability of the β-helix protein structure [74,75,76,77]. This bond’s degree of conformational freedom in interactions between protein surfaces is more rigid, although not as large as intra-protein interactions. Therefore, the analysis of these bindings is more inclined towards specificity for the binding site than TLR-3. Nevertheless, hydrogen bonds and salt bridges contribute to the stability of protein–protein interactions, but they do not bind specifically to the TLR-3 binding site. This interaction helps strengthen the bond between the spike glycoprotein surfaces, making it more difficult to separate [78].

### 3.8. Docking Study of the Constructed Vaccine Model against TLR-4

**Figure 5:**
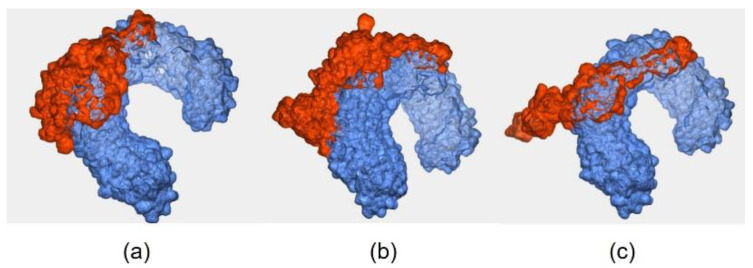
The docked complex of vaccine model of (**a**) EIJK-61453, (**b**) UGM0002, (**c**) B.1.1.7 with TLR-4.

All three vaccine models were successfully docked with TLR-4. The docked complex of the EIJK-61453-TLR-4 model had the most hydrogen bonds (15 hydrogen bonds) compared to the UGM0002-TLR-4 (13 hydrogen bonds) and B.1.1.7-TLR-4 models (12 hydrogen bonds). The complex of EIJK-61453-TLR-4, UGM0002-TLR-4, and B.1.1.7-TLR-4 also had two, three, and four salt bridge bonds, respectively. There was no significant difference among the docked complex vaccine–TLR-4 in terms of binding affinity, which was in the range of −16.3 to −15.5. Figure 5 shows the docking visualization of (a) EIJK-61453 (b) UGM0002 (c) B1.1.7 vaccine model against TLR-4. The overall interactions among vaccines model–TLR-4 are presented in Table 8.

### 3.9. Molecular Dynamics

**Figure 6:**
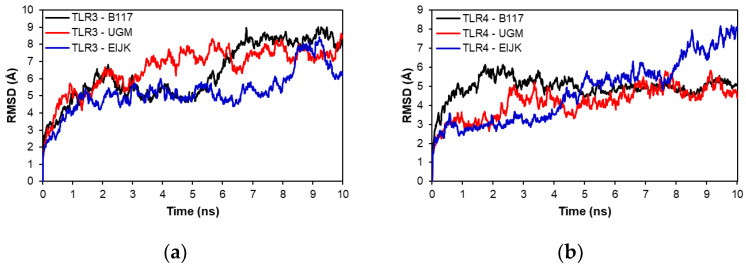
RMSD of TLR-3 (**a**) and TLR-4 (**b**) complexes.

**Figure 7:**
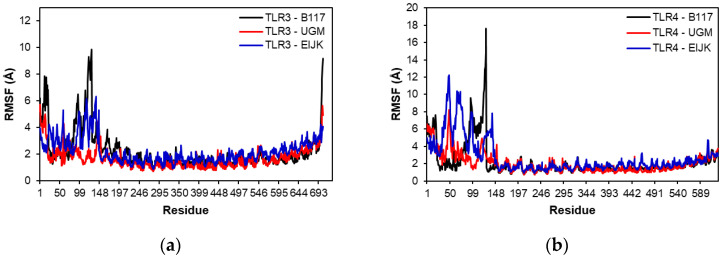
RMSF of TLR-3 (**a**) and TLR-4 (**b**) complexes.

Molecular dynamics analysis was performed to evaluate the stability of each vaccine-TLR complex model. Figure 6 shows the RMSD of the complexes in the 10 ns molecular dynamics simulation. As shown in Figure 6a, the UGM002 and EIJK vaccines showed an earlier stability time than B117 when interacting with TLR-3, although there were some fluctuations in the EIJK vaccine from 8 to 10 ns. In contrast, Figure 6b showed that the EIJK vaccine became stable at a later time than UGM002 and B117 vaccines when interacting with TLR-4. Furthermore, Figure 7 shows the RMSF of vaccine–TLR complex model. The interaction patterns with TLR-3 and TLR-4 were almost similar for all vaccine models. There were some fluctuations from the initial residue which lasted around the 100th residue. However, among the vaccine models, the B117 vaccine display the highest level of fluctuation.

### 3.10. In Silico Cloning and Immune Simulations

**Figure 8:**
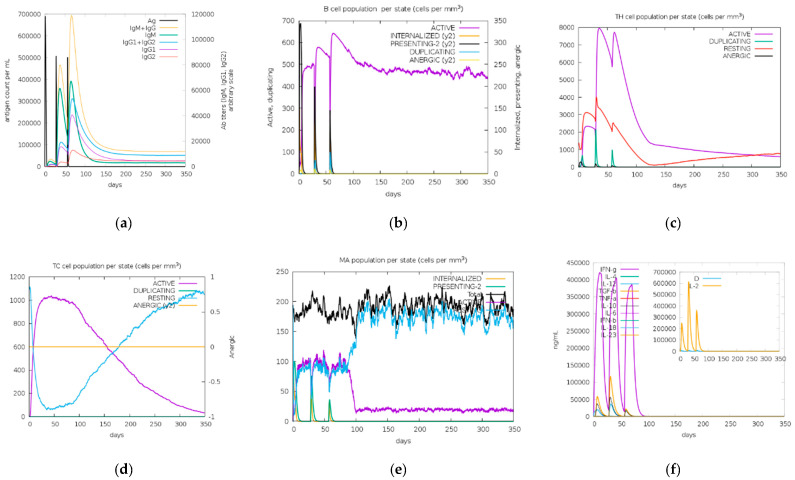
In silico immune simulation of EIJK-61453 vaccine using C-ImmSim server. Antigen and immunoglobulins (**a**), B cell population (**b**), TH cell population (**c**), TC cell population (**d**), macrophage population (**e**), production of cytokine and interleukins with Simpson index (**f**).

**Figure 9:**
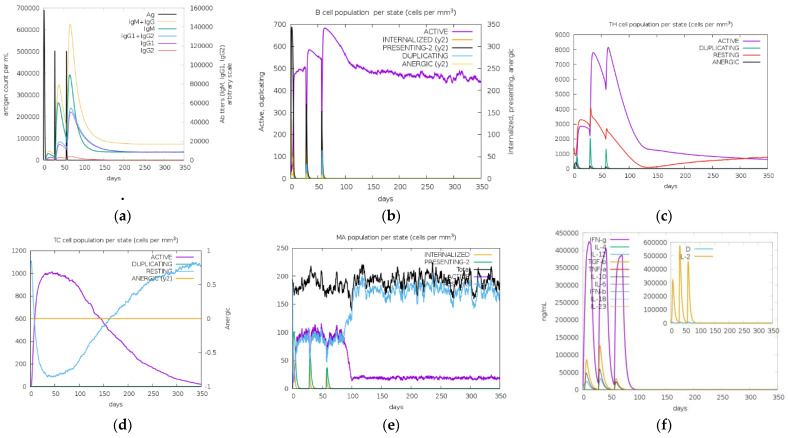
In silico immune simulation of UGM002 vaccine using the C-ImmSim server. Antigen and immunoglobulins (**a**), B cell population (**b**), TH cell population (**c**), TC cell population (**d**), macrophage population (**e**), production of cytokine and interleukins with Simpson index (**f**).

**Figure 10:**
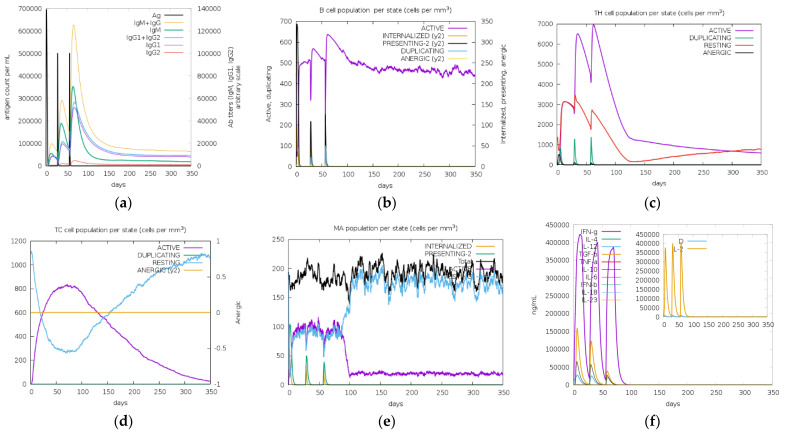
In silico immune simulation of the B.1.1.7 vaccine using C-ImmSim server. Antigen and immunoglobulins (**a**), B cell population (**b**), TH cell population (**c**), TC cell population (**d**), macrophage population (**e**), production of cytokine and interleukins with Simpson index (**f**).

From the immune simulation result (Figure 8, Figure 9 and Figure 10), three candidate vaccines (EIJK-61453, UGM0002, and B.1.1.7) showed similar patterns regarding the induction of immune cells (Figure 8, Figure 9 and Figure 10). The EIJK-61453 vaccine showed slightly higher IgM and IgG antibody responses after the second and tertiary injections than the other candidates, while the UGM0002 has the highest active B cell production. The immune responses from candidate vaccines were generally constructed from the initial injection and resulted in higher immune responses in the second and tertiary injections. This was followed by higher B cell and Th cell populations. The main distinct difference between the initial and the subsequent injections was the total population of memory cells. From the picture, it was clear that memory cells existence is higher after both secondary and tertiary injections than in the first one. Al Zamane et al. (2021) [79] stated that the next injection after the initial phase will activate B cells and T cells and they will create long-lasting protection and memory formation followed by antigen clearance during exposures. Furthermore, from the immune simulation result, innate and cytokine based immune regulation were also involved. Macrophages as an innate immune system are responsible for creating an inflammatory response to antigen exposure. Thus, its activation will release some cytokines such as IL-12 and IL-18 to stimulate adaptive immune response and IFN-gamma production to enhance immune activation [80,81]. Therefore, these candidates are capable of inducing good immune responses.

**Figure 11:**
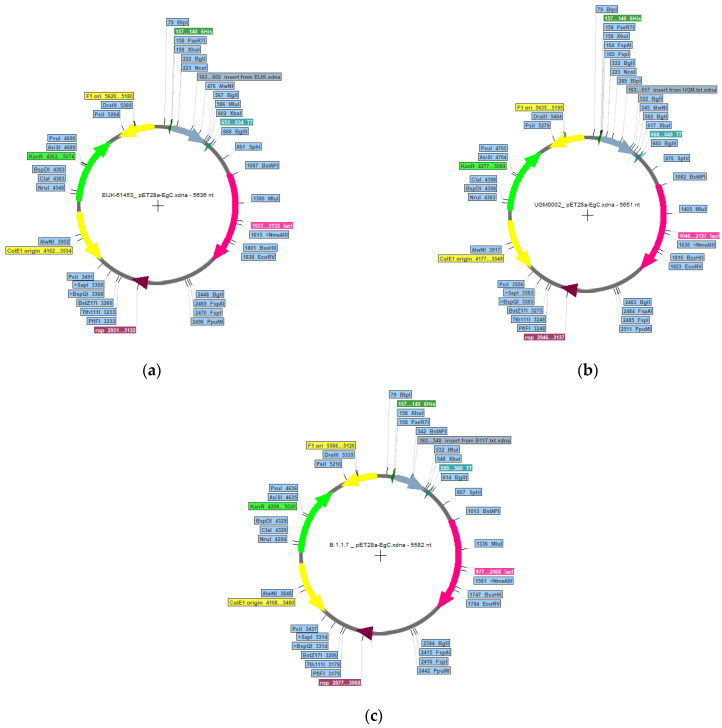
In silico cloning design of EIJK-61453 (**a**), UGM0002 (**b**), and B.1.1.7 (**c**) vaccine to pET28a-EgC vector. The vaccine sequence is shown by the blue-grey color arrow.

Alongside immune simulation, codon optimization was performed for the three vaccine candidates. In this work, the codon optimization used was based on *Escherichia coli* strain K12 expression systems. *E. coli* expression systems are well-known systems and still dominate in recombinant protein production. The use of *E. coli* as the recombinant vector has several advantages, including fast growth, ease of achieving high cell density, and the availability of components for rich complex media [82,83]. The important parameters for codon optimization are GC contents (30–70%), and a codon adaptation index (CAI) (>0.8). Table S6 showed that the %GC contents for all candidates were around 59% with CAI above 0.95. CAI has been used for the assessment of highly expressed genes, the closer the value of CAI to 1, the higher the chance of the genes being expressed [84]. From the results, %GC contents and CAI from vaccine candidates represent the ideal sequence for gene expression. To simulate vaccine expression, plasmid cloning vector pET28a-EgC was constructed with XbaI and XhoI as restriction sites. The plasmid constructions result revealed that EIJK-61453, UGM0002, and B.1.1.7 have 5636, 5652, and 5582 base pairs, respectively (Figure 11).

## 4. Conclusions

Our study has revealed a stable vaccine design that was created using the SARS-CoV-2 spike glycoprotein from Indonesia, which also became our limitation of the study. The evolution of the SARS-CoV-2 virus in Indonesia is not significantly different from its wild type, Wuhan-Hu-1, with the furthest similarity value of 99.84%. Several amino acid mutations occur and affect the construction of vaccine models. In this study, the sequences of EIJK-61453, UGM0002, and B.1.1.7 were chosen and used to search for CTL, HTL, and LBL epitopes. The selected epitopes from each sequence were then arranged with linkers and an adjuvant to construct a vaccine model. Among all the vaccine models, the UGM0002 vaccine is antigenic, non-allergenic, non-toxic, capable of inducing IFN-γ with a population reach of 86.29% in Indonesia, and activates the highest rate of B cells according to immune simulations. Furthermore, molecular dynamics analysis revealed that the interactions of the vaccines with TLR-3 or TLR-4 were stable. Therefore, the vaccine model of UGM0002 is recommended for further investigations.

## Figures and Tables

**Table 1:** HLA allele of the Indonesian Population used in the study.

Type of Analysis	HLA Allele
CTL	A*11:01, A*24:02, A*02:01, A*02:03, A*02:06, A*01:01, A*03:01, A*26: 01, A*30:01, A*32:01, B*15:02, B*44:03, B*18:01, B*58:01, B*40:01, B*51:01, B*35:03, B*57:01, B*07:02, and B*15:17
HTL	DRB1*12:02, DRB1*15:02, DRB1*07:01, DRB1*15:01, DRB1*03:01, DRB1*16:02, DRB1*09:01, DRB1*11:01, DRB1*04:05, DRB1*14:04, DRB1*10:01, DRB1*01:01, DRB1*13:02, DRB1*04:03, DRB1*04:02, DRB1*08:03, DRB1*14:01, DRB1*15:03, DRB1*12:01, DRB1*13:01, DRB1*08:02, DRB1*11:04, DPA1*01:03–DPB1*04:01, DPA1*02:01–DPB1*05:01, DPA1*01:03–DPB1*03:01, DPA1*01:03–DPB1*04:02, DPA1*01:03–DPB1*02:01, DPA1*02:01–DPB1*01:01, DPA1*02:01–DPB1*14:01, DQA1*06:01–DQB1*04:02, DQA1*01:01–DQB1*05:01, DQA1*01:02–DQB1*05:01, DQA1*01:02–DQB1*05:02, DQA1*01:02–DQB1*06:02, DQA1*02:01–DQB1*02:02, DQA1*02:01–DQB1*03:01, DQA1*02:01–DQB1*04:02, DQA1*03:01–DQB1*03:01, DQA1*03:01–DQB1*03:02, DQA1*01:03–DQB1*06:03, DQA1*05:01–DQB1*02:01, DQA1*05:01–DQB1*03:01, DQA1*05:01–DQB1*03:02, DQA1*05:01–DQB1*03:03, DQA1*05:01–DQB1*04:02, and DQA1*04:01–DQB1*04:02

**Table 2:** The list of amino acid mutations against the Wuhan-Hu-1 ((NC_045512.2:21563-25384).

	70	69	213	501	570	614	681	716	982	1118
Wuhan-Hu-1 (wild type)	V	H	V	N	A	D	P	T	S	D
EIJK-61453	V	H	V	N	A	D	P	T	S	D
UGM10002	V	H	A	N	A	G	P	T	S	D
B.1.1.7	-	-	V	Y	D	D	H	I	A	H

**Table 3:** Selected epitopes from the CTL analysis.

Sequence ID	CTL Epitope	HLA Allele	Percentile (%)	IC_50_ (nM)	Antigenicity	Allergenicity	Decision
Wuhan-Hu-1	SPRRARSVA	HLA-B*07:02	0.01	4.17	0.7729	N	Used
	KIYSKHTPI	HLA-A*32:01	0.02	10.98	0.7455	N	Used
		HLA-A*02:03	0.25	9.39			
	AEIRASANL	HLA-B*40:01	0.05	11.05	0.7082	N	Used
		HLA-B*44:03	0.12	71.39			
	QLTPTWRVY	HLA-B*15:02	0.03	62.69	1.2119	N	Used
	WTAGAAAYY	HLA-A*01:01	0.02	12.27	0.6306	N	Used
		HLA-A*26:01	0.03	11.63			
		HLA-B*15:17	0.04	2.6			
	IAIPTNFTI	HLA-B*51:01	0.06	302.84	0.7052	N	Used
		HLA-B*58:01	0.07	11.76			
		HLA-B*15:17	0.3	11.97			
	QYIKWPWYI	HLA-A*24:02	0.01	13.22	1.4177	Y	Not-used
	STQDLFLPF	HLA-A*32:01	0.04	17.27	0.6619	Y	Not-used
		HLA-A*26:01	0.3	437.88			
		HLA-B*15:17	0.5	26.56			
	TLLALHRS	HLA-B*15:02	0.05	98.39	0.7859	Y	Not-used
	YEQYIKWPW	HLA-B*18:01 HLA-B*44:03	0.01 0.05	4.19 38.77	0.869	Y	Not-used
EIJK-61453	SPRRARSVA	HLA-B*07:02	0.01	4.17	0.7729	N	Used
	KIYSKHTPI	HLA-A*32:01	0.02	10.98	0.7455	N	
		HLA-A*02:03	0.25	9.39		N	
	AEIRASANL	HLA-B*40:01 HLA-B*44:03	0.05 0.12	11.05 71.39	0.7082	N	
	QLTPTWRVY	HLA-B*15:02	0.03	62.69	1.2119	N	
	WTAGAAAYY	HLA-A*01:01	0.02	12.27	0.6306	N	
	IAIPTNFTI	HLA-A*26:01 HLA-B*15:17 HLA-B*51:01 HLA-B*58:01 HLA-B*15:17	0.03 0.04 0.06 0.07 0.3	11.63 2.6 302.84 11.76 11.97	0.7052	N	
	QYIKWPWYI STQDLFLPF TLLALHRS YEQYIKWPW	HLA-A*24:02 HLA-A*32:01 HLA-A*26:01 HLA-B*15:17 HLA-B*15:02 HLA-B*18:01 HLA-B*44:03	0.01 0.04 0.3 0.5 0.05 0.01 0.05	13.22 17.27 437.88 26.56 98.39 4.19 38.77	1.4177 0.6619 0.7859 0.869	Y	Not-used
UGM10002	SPRRARSVA KIYSKHTPI AEIRASANL GVYFASTEK QLTPTWRVY WTAGAAAYY IAIPTNFTI	HLA-B*07:02 HLA-A*32:01 HLA-A*02:03 HLA-B*40:01 HLA-B*44:03 HLA-A*11:01 HLA-A*03:01 HLA-A*30:01 HLA-B*15:02 HLA-A*01:01 HLA-A*26:01 HLA-B*15:17 HLA-B*51:01 HLA-B*58:01 HLA-B*15:17	0.01 0.02 0.25 0.05 0.12 0.07 0.12 0.5 0.03 0.02 0.03 0.04 0.06 0.07 0.3	4.17 10.98 9.39 11.05 71.39 15.17 23.87 98.99 62.69 12.27 11.63 2.6 302.84 11.76 11.97	0.7729 0.7455 0.7082 0.7112 1.2119 0.6306 0.7052	N	Used
	QYIKWPWYI STQDLFLPF ETKCTLKSF TLLALHRSY YEQYIKWPW	HLA-A*24:02 HLA-A*32:01 HLA-A*26:01 HLA-B*15:17 HLA-A*26:01 HLA-B*15:02 HLA-B*18:01 HLA-B*44:03	0.01 0.04 0.3 0.5 0.06 0.05 0.01 0.05	13.22 17.27 437.88 26.56 36.84 98.39 4.19 38.77	1.4177 0.6619 0.8720 0.8009 0.8690	Y	Not-used
B.1.1.7	KIYSKHTPI	HLA-A*32:01 HLA-A*02:03	0.02 0.25	10.98 9.39	0.7455	N	Used
	AEIRASANL	HLA-B*40:01 HLA-B*44:03	0.05 0.12	11.05 71.39	0.7082	N	Used
	WTAGAAAYY	HLA-A*01:01 HLA-A*26:01	0.02 0.03	12.27 11.63	0.6306	N	Used
	GVYFASTEK	HLA-B*15:17 HLA-A*11:01 HLA-A*03:01	0.04 0.07 0.12	2.6 15.17 23.87	0.6506	N	Used
	QLTPTWRVY	HLA-A*30:01 HLA-B*15:02	0.5 0.03	98.99 62.69	1.2119	N	Used
	QSYGFQPTY YEQYIKWPW IAIPINFTI STQDLFLPF ETKCTLKSF TLLALHRSY IPINFTISV	HLA-B*15:17 HLA-B*58:01 HLA-B*15:02 HLA-B*18:01 HLA-B*44:03 HLA-B*58:01 HLA-A*11:01 HLA-B*15:17 HLA-A*32:01 HLA-A*26:01 HLA-B*15:17 HLA-A*26:01 HLA-B*15:02 HLA-B*51:01 HLA-B*07:02	0.03 0.17 0.4 0.01 0.05 0.06 0.07 0.5 0.04 0.3 0.5 0.06 0.05 0.06 0.4	2.2 30.54 489.26 4.19 38.77 10.26 376.73 30 17.27 437.88 26.56 36.84 98.39 296.2 91.82	1.1150 0.8690 1.5131 0.6619 0.8720 0.8009 1.7137	Y	Not-used

**Table 4:** Selected epitopes from the HTL analysis.

Epitope	Seq. Position	Percentile (%)	Interaction with Allele HLA	IC_50_ (nM)	Antigenicity	Allergenicity	IFN-γ	Decision
Wuhan Hu-1								
GINITRFQTLLALHR	232–246	0.02	HLA-DRB1*04:02	96.22	0.5582	N	+	Used
		0.03	HLA-DRB1*15:01	10.96				
		0.04	HLA-DRB1*04:03	182.16				
HWFVTQRNFYEPQII CTFEYVSQPFLMDLE	1101–1115 166–180	0.04 0.06	HLA-DRB1*04:05 HLA-DPA1*01:03-	19.97 12.76	0.5225 0.5700		− +	
			DPB1*04:01					
RFQTLLALHRSYLTP	237–251	0.07	HLA-DRB1*14:01	66.04	0.5470		−	
IIAYTMSLGAENSV	692–705	0.07	HLA-DRB1*10:01	7.8	0.5350		−	
KTQSLLIVNNATNVV	113–127	0	HLA-DRB1*13:02	6.37	0.6303	Y		Not-used
AAEIRASANLAATKM	1015–1029	0.03	HLA-DRB1*04:02	101.18	0.7125			
NCTFEYVSQPFLMDL	165–179	0.07 0.07	HLA-DRB1*04:03 HLA-DPA1*01:03-DPB1*04:01	220.76 12.96	0.5206			
AEIRASANLAATKMS	1016–1030	0.07	HLA-DRB1*04:02	122.53	0.8255			
PINLVRDLPQGFSAL	209–223	0.07	HLA-DRB1*03:01	27.42	0.6086			
EIJK-61453								
GINITRFQTLLALHR	232–246	0.02	HLA-DRB1*04:02	96.22	0.5582		+	Used
		0.03	HLA-DRB1*15:01	10.96				
		0.04	HLA-DRB1*04:03	182.16				
HWFVTQRNFYEPQII CTFEYVSQPFLMDLE	1101–1115 166–180	0.04 0.06	HLA-DRB1*04:05 HLA-DPA1*01:03-	19.97 12.76	0.5225 0.5700	N	− +	
			DPB1*04:01					
RFQTLLALHRSYLTP	237–251	0.07	HLA-DRB1*14:01	66.04	0.5470		−	
IIAYTMSLGAENSVA	692–705	0.07	HLA-DRB1*10:01	7.8	0.5350		−	
KTQSLLIVNNATNVV	113–127	0	HLA-DRB1*13:02	6.37	0.6303			Not-used
AAEIRASANLAATKM NCTFEYVSQPFLMDL	1015–1029 165–179	0.03 0.07 0.07	“HLA-DRB1*04:02 HLA-DRB1*04:03 HLA-DPA1*01:03-	101.18 220.76 12.96	0.7125 0.5206	Y		
AEIRASANLAATKMS PINLVRDLPQGFSAL	1016–1030 209–223	0.07 0.07	DPB1*04:01 HLA-DRB1*04:02 HLA-DRB1*03:01	122.53 27.42	0.8255 0.6086			
UGM10002								
GINIFQTLLALHRTR HWFVTQRNFYEPQII CTFEYVSQPFLMDLE NCTFEYVSQPFLMDL	232–246 1101–1115 166–180 165–179	0.02 0.03 0.04 0.04 0.06 0.07	HLA-DRB1*04:02 HLA-DRB1*15:01 HLA-DRB1*04:03” HLA-DRB1*04:05 HLA-DPA1*01:03- DPB1*04:01 HLA-DPA1*01:03- DPB1*04:01	96.22 10.96 182.16 19.97 12.76 12.96	0.5582 0.5225 0.5700 0.5206	N	+ − + −	Used
KTQSLLIVNNATNVV AAEIRASANLAATKM IIAYTMSLGAENSVA AEIRASANLAATKMS	113–127 1015–1029 692–705 1016–1030	0 0.03 0.07 0.07 0.07	HLA-DRB1*13:02 “HLA-DRB1*04:02 HLA-DRB1*04:03” HLA-DRB1*10:01 HLA-DRB1*04:02	6.37 101.18 220.76 7.8 122.53	0.6303 0.7125 0.5426 0.8255	Y		Not-used
B.1.1.7								
RAAEIRASANLAATK KHTPINLVRDLPQGF KGIYQTSNFRVQPTE	1014–1028 206–220 310–324	0.08 0.08 0.07	HLA-DRB1*04:02 HLA-DRB1*04:02 HLA-DPA1*02:01- DPB1*05:01	125.96 193.31 1110.1	0.5709 0.5644 0.8838	Non-Al Non-Al Non-Al	+ − +	Used
TGCVIAWNSNNLDSK EKGIYQTSNFRVQPT VEKGIYQTSNFRVQP AAEIRASANLAATKM NDPFLGVYYHKNNKS CNDPFLGVYYHKNNK	430–444 309–323 308–322 1015–1029 137–151 136–150	0.06 0.07 0.09 0.03 0.07 0.02 0.03	HLA-DRB1*15:01 HLA-DRB1*15:02 HLA-DRB1*15:02 HLA-DRB1*04:02, HLA-DRB1*04:03 HLA-DPA1*02:01- DPB1*05:01 HLA-DPA1*02:01- DPB1*05:01	30.1 84.33 86.62 101.18 220.76 923.7 994.78	0.6531 0.9243 0.7959 0.7637 0.8199 0.6472	Al Al Al Al Al Al		Not-used

+ means capable to stimulate IFN-γ.

**Table 5:** Predicted results of LBL epitope for each sequence.

Sequence ID	Epitope	Antigenicity	IFN-γ
Wuhan-Hu-1	NNLDSKVGGNYNY	0.9437	+
	FQPTNG	0.7429	+
	AYTMSLGAENSVAYSN	0.6003	+
	GQSKRVDFC	1.779	+
	SCCKFDEDDSEPVLKGVKL	0.6085	+
EIJK-61453	NNLDSKVGGNYNY	0.9437	+
	FQPTNG	0.7429	+
	AYTMSLGAENSVAYSN	0.6003	+
	GQSKRVDFC	1.779	+
	SCCKFDEDDSEPVLKGVKL	0.6085	+
UGM0002	NNLDSKVGGNYNY	0.9437	+
	SNKKFLPF	1.3952	+
	VNCTEV	0.6529	+
	TNTSNQ	1.6803	+
	LTPTWRVYSTGSNVFQT	0.5474	+
	GQSKRVDFC	1.779	+
B.1.1.7	GDEVRQIAPGQTGKIA	1.0202	+
	SNKKFLPF	1.3952	+
	VNCTEV	1.6803	+
	LGQSKRVDFC	1.8685	+
	SCCKFDEDDSEPVLKGVK	0.5409	+

**Table 6:** List of the best multi-epitope vaccines for each sequence.

Vaccine Sequences	Score	Explanation
Wuhan-Hu-1		
MRIHYLLFALLFLFLVPVPGHGGIINTLQKYYCRVRGGRCAVLSCLPKEEQIGKCSTRGRKCCRRKKEAAAKSPRRARSVAKKWTAGAAAYYGPGPGGINITRFQTLLALHRGPGPGCTFEYVSQPFLMDLEGPGPGGQSKRVDFC	Quality Factor ERRAT = 91.73 TM-Score = 0.383 Template = 1KJ6_A Confidence = 97.0%	Using the template protein 1KJ6_A with 97.0% confidence Vaccine length 146 aa
EIJK-61453		
MRIHYLLFALLFLFLVPVPGHGGIINTLQKYYCRVRGGRCAVLSCLPKEEQIGKCSTRGRKCCRRKKEAAAKSPRRARSVAKKWTAGAAAYYGPGPGGINITRFQTLLALHRGPGPGCTFEYVSQPFLMDLEGPGPGGQSKRVDFC	Quality Factor ERRAT = 91.73 TM-Score = 0.383 Template = 1KJ6_A Confidence = 97.0%	Using the template protein 1KJ6_A with 97.0% confidence Vaccine length 146 aa
UGM0002		
MRIHYLLFALLFLFLVPVPGHGGIINTLQKYYCRVRGGRCAVLSCLPKEEQIGKCSTRGRKCCRRKKEAAAKSPRRARSVAAAYKIYSKHTPIAAYGVYFASTEKAAYWTAGAAAYYGPGPGGINIFQTLLALHRTRGPGPGGQSKRVDFC	Quality Factor ERRAT = 99.27 TM-Score = 0.358	Using a de novo folding approach Vaccine length 151 aa
B.1.1.7		
MRIHYLLFALLFLFLVPVPGHGGIINTLQKYYCRVRGGRCAVLSCLPKEEQIGKCSTRGRKCCRRKKEAAAKKIYSKHTPIAAYWTAGAAAYYGPGPGRAAEIRASANLAATKGPGPGLGQSKRVDFC	Quality Factor ERRAT = 97.32 TM-Score = 0.405 Template = 6VSJ_C Confidence = 100%	Using the 6VSJ_C protein template with 100% confidence Vaccine length 128 aa

**Table 7:** The bond type and amino acid residues interacting between vaccine models and TLR-3.

Vaccine Model	Binding Affinity ΔG (kcal/mol)	RMSD	Interaction with TLR-3
EIJK-61453 Total Interactions: 18 hydrogen bonds 3 salt bridges 210 nonbonded contacts	−18.7	0.5 ± 0.3	Hydrogen bond: Vaccine Residues—TLR-3 TYR5-ASP575 (2.65 Å), LEU15-HIS539 (2.70 Å), PRO17-ARG489 (2.86 Å), VAL18-ARG489 (3.10 Å), GLY22-ARG488 (2.60 Å), GLU50-ARG331 (2.67 Å), GLN51-LYS335 (3.00 Å), LYS54-ASN388 (2.65 Å), LYS54-LYS416 (2.78 Å), ARG58-LYS418 (2.70 Å), ARG78-ASN257 (2.73 Å), ARG78-GLN259 (2.89 Å), THR85-TYR307 (2.71 Å), TYR91-LYS416 (2.68 Å), LEU131-GLN618 (2.76 Å), GLU132-LYS619 (2.60 Å), GLY133-GLU570 (3.25 Å), GLN139-ASP536 (2.64 Å) Salt bridges: GLU 50-ARG331 (2.67 Å), LYS 61-ASP366 (3.56 Å), GLU132-LYS619 (2.60 Å)
UGM0002 Total Interactions: 11 hydrogen bonds 3 salt bridges 218 nonbonded contacts	−19.4	12.4 ± 0.2	Hydrogen bond: Vaccine Residues—TLR-3 PHE14-HIS563 (2.69 Å), HIS21-HIS565 (2.73 Å), GLY23-GLU533 (2.72 Å), ASN26-GLU533 (2.73 Å), GLN51-TYR326 (2.77 Å), LYS67-GLU358 (2.88 Å), ALA71-ARG251 (2.88 Å), LYS72-GLU301 (3.24 Å), ILE124-ASN247 (2.75 Å), GLN128-TRP273 (2.85 Å), ARG135-ALA295 (2.78 Å)
B.1.1.7 Total Interactions: 12 hydrogen bonds 4 salt bridges 138 non-bonded contacts	−15.6	2.3 ± 0.5	Hydrogen bond: Vaccine Residues—TLR-3 PHE14-TYR307 (2.95 Å), VAL16-LYS330 (2.61 Å), HIS21-GLU363 (2.58 Å), ARG36-GLU533 (2.92 Å), LYS54-LYS382 (3.11 Å), LYS54-HIS410 (2.94 Å), CYS55-TYR383 (2.90 Å), SER56-TYR462 (3.18 Å), THR57-TYR383 (2.78 Å), ARG64-GLU533 (2.77 Å), LYS72-GLU533 (2.70 Å), TYR84-GLU587 (2.61 Å) Salt bridges: HIS 21-GLU363 (2.58 Å), ARG 36-GLU533 (2.92 Å), ARG 64-GLU533 (2.77 Å), LYS 72-GLU533 (2.70 Å)

**Table 8:** The bond type and amino acid residues interacting between vaccine models and TLR-4.

Vaccine Model	Binding Affinity ΔG (kcal/mol)	RMSD	Interaction with TLR-4
EIJK-61453 Total Interactions: 15 hydrogen bonds 2 salt bridges 137 nonbonded contacts	−16.3	1.1 ± 0.3	Hydrogen bond: Vaccine Residues—TLR-4 ARG34-ARG264 (2.79 Å), PRO116-ARG264 (2.77 Å), ARG64-ASN265 (2.68 Å), GLU68-ASN265 (3.04 Å), ARG76-GLU266 (2.76 Å), CYS118-TYR296 (3.23 Å), CYS146-LYS341 (2.98 Å), HIS21-HIS41 (2.84 Å), LEU13-ASN433 (2.84 Å), ALA9-LYS435 (2.73 Å), ARG2-GLU439 (2.60 Å) Salt bridges: LYS72-GLU266 (2.98 Å), ARG2-GLU439 (2.60 Å)
UGM0002 Total Interactions: 13 hydrogen bonds 3 salt bridges 170 nonbonded contacts	−16.1	1.0 ± 0.3	Hydrogen bond: Vaccine Residues—TLR-3 ARG2-GLU270 (2.95 Å), ARG2-ASP294 (2.55 Å), LEU6-TYR296 (2.68 Å), ARG34-PHE387 (2.97 Å), ARG39-LYS388 (3.20 Å), ARG34-LYS399 (3.20 Å), ARG39-GLN436 (2.98 Å), LYS105-GLU439 (3.22 Å), LYS66-GLU439 (2.60 Å), ARG147-ASP490 (2.59 Å) Salt bridges: ARG2-ASP294 (2.55 Å), LYS66-GLU439 (2.60 Å), ARG147-ASP490 (2.59 Å)
B.1.1.7 Total Interactions: 12 hydrogen bonds 4 salt bridges 162 nonbonded contacts	−15.5	26.4 ± 0.2	Hydrogen bond: Vaccine Residues—TLR-3 ALA9-ARG264 (2.79 Å), ARG2-ASN264 (2.68 Å), ARG2-GLU266 (3.04 Å), ARG64-GLU439 (2.60 Å), GLN51-ARG460 (2.85 Å), GLU50-ARG460 (2.72 Å), GLN51-ARG460 (3.12 Å), GLU50-GLN484 (3.19 Å), LYS66-GLU485 (2.66 Å) Salt bridges: ARG2-GLU266 (2.64 Å), ARG64-GLU439 (2.60 Å), GLU50-ARG460 (2.72 Å), LYS66-GLU485 (2.66 Å)

## Data Availability

Not applicable.

## Supplementary Materials

The following supporting information can be downloaded at: https://www.mdpi.com/article/10.3390/vaccines11020399/s1, Figure S1: Phylogenetic Tree Results from: (a) Neighbor-Joining Method (b) Maximum Likelihood Method; Figure S2: Number of epitopes recognized by HLA from Indonesia population for Wuhan-Hu-1 (a), EIJK-61453 (b), UGM0002 (c), and B.1.1.7 (d); Figure S3: Ramachandran plot for (a) Wuhan-Hu-1-2 and EIJK-61453-2 with 454 aa, 88.2% in the most-favorable region, 59 Glycine residues, and 38 Proline residues; Figure S4: 3D structure prediction of (a) Wuhan-Hu-1-2 and EIJK-61453-2 vaccine, (b) Wuhan-Hu-1-3 and EIJK-61453-3 vaccine (c) UGM0002-2, (d) UGM0002-3, (e) B.1.1.7-1-1, and (e) B.1.1.7-1-3; Table S1: Wuhan-Hu-1 and EIJK-61453 epitopes population coverage; Table S2: UGM0002 epitopes population coverage; Table S3: B.1.1.7 epitopes population coverage; Table S4: The predicted discontinuous B-cell epitopes; Table S5: Top three list of vaccine construction sequences; Table S6: Optimized vaccine sequences.
