# Supplementary material for: Immunoinformatics Study: Multi-Epitope Based Vaccine Design from SARS-CoV-2 Spike Glycoprotein
Source: Vaccines (Basel). 2023 Feb 9;11(2):399. doi: 10.3390/vaccines11020399 (PMC9964839; doi:10.3390/vaccines11020399)
Supplement: Supplementary file 1 [file vaccines-11-00399-s001.zip › vaccines-2119457-supplementary.pdf]

Supplementary

Immunoinformatics Study: Multi-Epitope Based Vaccine Design from SARS-CoV-2 Spike Glycoprotein in Indonesia

Ramadhita Umitaibatin[1], Azza Hanif Harisna[2], Muhammad Miftah Jauhar[2], Putri Hawa Syaifie[2], Adzani Gaisani Arda[2], Dwi Wahyu Nugroho[2], Donny Ramadhan[3], Etik Mardliyati[4], Wervyan Shalannanda[5], and Isa Anshori[1,*]

[1] Lab-on-chip Group, Department of Biomedical Engineering, School of Electrical Engineering and Informatics, Bandung Institute of Technology, Bandung, Jawa Barat 40132, Indonesia
[2] Nano Center Indonesia, Jl. PUSPIPTEK, South Tangerang, Banten, 15314, Indonesia
[3] Research Center for Pharmaceutical Ingredients and Traditional Medicine, National Research and Innovation Agency (BRIN), Cibinong, Bogor, Jawa Barat, 16911, Indonesia
[4] Research Center for Vaccine and Drug, National Research and Innovation Agency (BRIN), Cibinong, Bogor, Jawa Barat, 16911, Indonesia
[5] Department of Telecommunication Engineering, School of Electrical Engineering and Informatics, Bandung Institute of Technology, Bandung, Jawa Barat 40132, Indonesia
* Correspondence: isaa@staff.stei.itb.ac.id

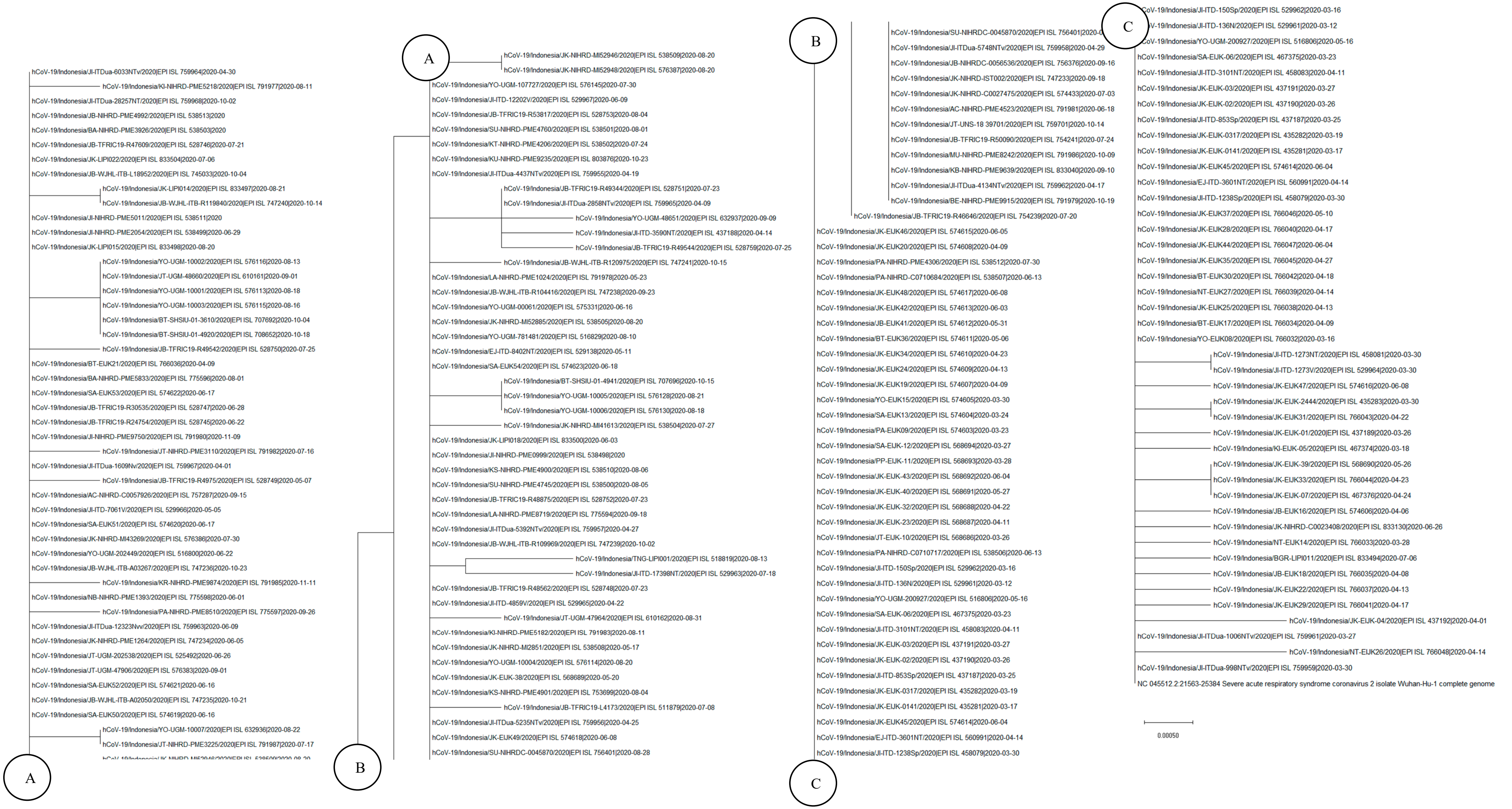
A
hCoV-19/Indonesia/JI-ITDua-6033NTv/2020|EPI ISL 759964|2020-04-30
hCoV-19/Indonesia/KI-NIHRD-PME5218/2020|EPI ISL 791977|2020-08-11
hCoV-19/Indonesia/JI-ITDua-28257NT/2020|EPI ISL 759968|2020-10-02
hCoV-19/Indonesia/JB-NIHRD-PME4992/2020|EPI ISL 538513|2020
hCoV-19/Indonesia/BA-NIHRD-PME3926/2020|EPI ISL 538503|2020
hCoV-19/Indonesia/JB-TFRIC19-R47609/2020|EPI ISL 528746|2020-07-21
hCoV-19/Indonesia/JK-LIPI022/2020|EPI ISL 833504|2020-07-06
hCoV-19/Indonesia/JB-WJHL-ITB-L18952/2020|EPI ISL 745033|2020-10-04
hCoV-19/Indonesia/JK-LIPI014/2020|EPI ISL 833497|2020-08-21
hCoV-19/Indonesia/JB-WJHL-ITB-R119840/2020|EPI ISL 747240|2020-10-14
hCoV-19/Indonesia/JI-NIHRD-PME5011/2020|EPI ISL 538511|2020
hCoV-19/Indonesia/JI-NIHRD-PME2054/2020|EPI ISL 538499|2020-06-29
hCoV-19/Indonesia/JK-LIPI015/2020|EPI ISL 833498|2020-08-20
hCoV-19/Indonesia/YO-UGM-10002/2020|EPI ISL 576116|2020-08-13
hCoV-19/Indonesia/JT-UGM-48660/2020|EPI ISL 610161|2020-09-01
hCoV-19/Indonesia/YO-UGM-10001/2020|EPI ISL 576113|2020-08-18
hCoV-19/Indonesia/YO-UGM-10003/2020|EPI ISL 576115|2020-08-16
hCoV-19/Indonesia/BT-SHSIU-01-3610/2020|EPI ISL 707692|2020-10-04
hCoV-19/Indonesia/BT-SHSIU-01-4920/2020|EPI ISL 708652|2020-10-18
hCoV-19/Indonesia/JB-TFRIC19-R49542/2020|EPI ISL 528750|2020-07-25
hCoV-19/Indonesia/BT-EIJK21/2020|EPI ISL 766036|2020-04-09
hCoV-19/Indonesia/BA-NIHRD-PME5833/2020|EPI ISL 775596|2020-08-01
hCoV-19/Indonesia/SA-EIJK53/2020|EPI ISL 574622|2020-06-17
hCoV-19/Indonesia/JB-TFRIC19-R30535/2020|EPI ISL 528747|2020-06-28
hCoV-19/Indonesia/JB-TFRIC19-R24754/2020|EPI ISL 528745|2020-06-22
hCoV-19/Indonesia/JI-NIHRD-PME9750/2020|EPI ISL 791980|2020-11-09
hCoV-19/Indonesia/JT-NIHRD-PME3110/2020|EPI ISL 791982|2020-07-16
hCoV-19/Indonesia/JI-ITDua-1609Nv/2020|EPI ISL 759967|2020-04-01
hCoV-19/Indonesia/JB-TFRIC19-R4975/2020|EPI ISL 528749|2020-05-07
hCoV-19/Indonesia/AC-NIHRD-C0057926/2020|EPI ISL 757287|2020-09-15
hCoV-19/Indonesia/JI-ITD-7061V/2020|EPI ISL 529966|2020-05-05
hCoV-19/Indonesia/SA-EIJK51/2020|EPI ISL 574620|2020-06-17
hCoV-19/Indonesia/JK-NIHRD-MI43269/2020|EPI ISL 576386|2020-07-30
hCoV-19/Indonesia/YO-UGM-202449/2020|EPI ISL 516800|2020-06-22
hCoV-19/Indonesia/JB-WJHL-ITB-A03267/2020|EPI ISL 747236|2020-10-23
hCoV-19/Indonesia/KR-NIHRD-PME9874/2020|EPI ISL 791985|2020-11-11
hCoV-19/Indonesia/NB-NIHRD-PME1393/2020|EPI ISL 775598|2020-06-01
hCoV-19/Indonesia/PA-NIHRD-PME8510/2020|EPI ISL 775597|2020-09-26
hCoV-19/Indonesia/JI-ITDua-12323Nvv/2020|EPI ISL 759963|2020-06-09
hCoV-19/Indonesia/JK-NIHRD-PME1264/2020|EPI ISL 747234|2020-06-05
hCoV-19/Indonesia/JT-UGM-202538/2020|EPI ISL 525492|2020-06-26
hCoV-19/Indonesia/JT-UGM-47906/2020|EPI ISL 576383|2020-09-01
hCoV-19/Indonesia/SA-EIJK52/2020|EPI ISL 574621|2020-06-16
hCoV-19/Indonesia/JB-WJHL-ITB-A02050/2020|EPI ISL 747235|2020-10-21
hCoV-19/Indonesia/SA-EIJK50/2020|EPI ISL 574619|2020-06-16
hCoV-19/Indonesia/YO-UGM-10007/2020|EPI ISL 632936|2020-08-22
hCoV-19/Indonesia/JT-NIHRD-PME3225/2020|EPI ISL 791987|2020-07-17
A
B
hCoV-19/Indonesia/JK-NIHRD-MI52946/2020|EPI ISL 538509|2020-08-20
hCoV-19/Indonesia/JK-NIHRD-MI52948/2020|EPI ISL 576387|2020-08-20
hCoV-19/Indonesia/YO-UGM-107727/2020|EPI ISL 576145|2020-07-30
hCoV-19/Indonesia/JI-ITD-12202V/2020|EPI ISL 529967|2020-06-09
hCoV-19/Indonesia/JB-TFRIC19-R53817/2020|EPI ISL 528753|2020-08-04
hCoV-19/Indonesia/SU-NIHRD-PME4760/2020|EPI ISL 538501|2020-08-01
hCoV-19/Indonesia/KT-NIHRD-PME4206/2020|EPI ISL 538502|2020-07-24
hCoV-19/Indonesia/KU-NIHRD-PME9235/2020|EPI ISL 803876|2020-10-23
hCoV-19/Indonesia/JI-ITDua-4437NTv/2020|EPI ISL 759955|2020-04-19
hCoV-19/Indonesia/JB-TFRIC19-R49344/2020|EPI ISL 528751|2020-07-23
hCoV-19/Indonesia/JI-ITDua-2858NTv/2020|EPI ISL 759965|2020-04-09
hCoV-19/Indonesia/YO-UGM-48651/2020|EPI ISL 632937|2020-09-09
hCoV-19/Indonesia/JI-ITD-3590NT/2020|EPI ISL 437188|2020-04-14
hCoV-19/Indonesia/JB-TFRIC19-R49544/2020|EPI ISL 528759|2020-07-25
hCoV-19/Indonesia/JB-WJHL-ITB-R120975/2020|EPI ISL 747241|2020-10-15
hCoV-19/Indonesia/LA-NIHRD-PME1024/2020|EPI ISL 791978|2020-05-23
hCoV-19/Indonesia/JB-WJHL-ITB-R104416/2020|EPI ISL 747238|2020-09-23
hCoV-19/Indonesia/YO-UGM-00061/2020|EPI ISL 575331|2020-06-16
hCoV-19/Indonesia/JK-NIHRD-MI52885/2020|EPI ISL 538505|2020-08-20
hCoV-19/Indonesia/YO-UGM-781481/2020|EPI ISL 516829|2020-08-10
hCoV-19/Indonesia/EJ-ITD-8402NT/2020|EPI ISL 529138|2020-05-11
hCoV-19/Indonesia/SA-EIJK54/2020|EPI ISL 574623|2020-06-18
hCoV-19/Indonesia/BT-SHSIU-01-4941/2020|EPI ISL 707696|2020-10-15
hCoV-19/Indonesia/YO-UGM-10005/2020|EPI ISL 576128|2020-08-21
hCoV-19/Indonesia/YO-UGM-10006/2020|EPI ISL 576130|2020-08-18
hCoV-19/Indonesia/JK-NIHRD-MI41613/2020|EPI ISL 538504|2020-07-27
hCoV-19/Indonesia/JK-LIPI018/2020|EPI ISL 833500|2020-06-03
hCoV-19/Indonesia/JI-NIHRD-PME0999/2020|EPI ISL 538498|2020
hCoV-19/Indonesia/KS-NIHRD-PME4900/2020|EPI ISL 538510|2020-08-06
hCoV-19/Indonesia/SU-NIHRD-PME4745/2020|EPI ISL 538500|2020-08-05
hCoV-19/Indonesia/JB-TFRIC19-R48875/2020|EPI ISL 528752|2020-07-23
hCoV-19/Indonesia/LA-NIHRD-PME8719/2020|EPI ISL 775594|2020-09-18
hCoV-19/Indonesia/JI-ITDua-5392NTv/2020|EPI ISL 759957|2020-04-27
hCoV-19/Indonesia/JB-WJHL-ITB-R109969/2020|EPI ISL 747239|2020-10-02
hCoV-19/Indonesia/TNG-LIPI001/2020|EPI ISL 518819|2020-08-13
hCoV-19/Indonesia/JI-ITD-17398NT/2020|EPI ISL 529963|2020-07-18
hCoV-19/Indonesia/JB-TFRIC19-R48562/2020|EPI ISL 528748|2020-07-23
hCoV-19/Indonesia/JI-ITD-4859V/2020|EPI ISL 529965|2020-04-22
hCoV-19/Indonesia/JT-UGM-47964/2020|EPI ISL 610162|2020-08-31
hCoV-19/Indonesia/KI-NIHRD-PME5182/2020|EPI ISL 791983|2020-08-11
hCoV-19/Indonesia/JK-NIHRD-MI2851/2020|EPI ISL 538508|2020-05-17
hCoV-19/Indonesia/YO-UGM-10004/2020|EPI ISL 576114|2020-08-20
hCoV-19/Indonesia/JK-EIJK-38/2020|EPI ISL 568689|2020-05-20
hCoV-19/Indonesia/KS-NIHRD-PME4901/2020|EPI ISL 753699|2020-08-04
hCoV-19/Indonesia/JB-TFRIC19-L4173/2020|EPI ISL 511879|2020-07-08
hCoV-19/Indonesia/JI-ITDua-5235NTv/2020|EPI ISL 759956|2020-04-25
hCoV-19/Indonesia/JK-EIJK49/2020|EPI ISL 574618|2020-06-08
hCoV-19/Indonesia/SU-NIHRDC-0045870/2020|EPI ISL 756401|2020-08-28
B
hCoV-19/Indonesia/SU-NIHRDC-0045870/2020|EPI ISL 756401|2020-0
hCoV-19/Indonesia/JI-ITDua-5748NTv/2020|EPI ISL 759958|2020-04-29
hCoV-19/Indonesia/JB-NIHRDC-0056536/2020|EPI ISL 756376|2020-09-16
hCoV-19/Indonesia/JK-NIHRD-IST002/2020|EPI ISL 747233|2020-09-18
hCoV-19/Indonesia/JK-NIHRD-C0027475/2020|EPI ISL 574433|2020-07-03
hCoV-19/Indonesia/AC-NIHRD-PME4523/2020|EPI ISL 791981|2020-06-18
hCoV-19/Indonesia/JT-UNS-18 39701/2020|EPI ISL 759701|2020-10-14
hCoV-19/Indonesia/JB-TFRIC19-R50090/2020|EPI ISL 754241|2020-07-24
hCoV-19/Indonesia/MU-NIHRD-PME8242/2020|EPI ISL 791986|2020-10-09
hCoV-19/Indonesia/KB-NIHRD-PME9639/2020|EPI ISL 833040|2020-09-10
hCoV-19/Indonesia/JI-ITDua-4134NTv/2020|EPI ISL 759962|2020-04-17
hCoV-19/Indonesia/BE-NIHRD-PME9915/2020|EPI ISL 791979|2020-10-19
hCoV-19/Indonesia/JB-TFRIC19-R46646/2020|EPI ISL 754239|2020-07-20
hCoV-19/Indonesia/JK-EIJK46/2020|EPI ISL 574615|2020-06-05
hCoV-19/Indonesia/JK-EIJK20/2020|EPI ISL 574608|2020-04-09
hCoV-19/Indonesia/PA-NIHRD-PME4306/2020|EPI ISL 538512|2020-07-30
hCoV-19/Indonesia/PA-NIHRD-C0710684/2020|EPI ISL 538507|2020-06-13
hCoV-19/Indonesia/JK-EIJK48/2020|EPI ISL 574617|2020-06-08
hCoV-19/Indonesia/JK-EIJK42/2020|EPI ISL 574613|2020-06-03
hCoV-19/Indonesia/JB-EIJK41/2020|EPI ISL 574612|2020-05-31
hCoV-19/Indonesia/BT-EIJK36/2020|EPI ISL 574611|2020-05-06
hCoV-19/Indonesia/JK-EIJK34/2020|EPI ISL 574610|2020-04-23
hCoV-19/Indonesia/JK-EIJK24/2020|EPI ISL 574609|2020-04-13
hCoV-19/Indonesia/JK-EIJK19/2020|EPI ISL 574607|2020-04-09
hCoV-19/Indonesia/YO-EIJK15/2020|EPI ISL 574605|2020-03-30
hCoV-19/Indonesia/SA-EIJK13/2020|EPI ISL 574604|2020-03-24
hCoV-19/Indonesia/PA-EIJK09/2020|EPI ISL 574603|2020-03-23
hCoV-19/Indonesia/SA-EIJK-12/2020|EPI ISL 568694|2020-03-27
hCoV-19/Indonesia/PP-EIJK-11/2020|EPI ISL 568693|2020-03-28
hCoV-19/Indonesia/JK-EIJK-43/2020|EPI ISL 568692|2020-06-04
hCoV-19/Indonesia/JK-EIJK-40/2020|EPI ISL 568691|2020-05-27
hCoV-19/Indonesia/JK-EIJK-32/2020|EPI ISL 568688|2020-04-22
hCoV-19/Indonesia/JK-EIJK-23/2020|EPI ISL 568687|2020-04-11
hCoV-19/Indonesia/JT-EIJK-10/2020|EPI ISL 568686|2020-03-26
hCoV-19/Indonesia/PA-NIHRD-C0710717/2020|EPI ISL 538506|2020-06-13
hCoV-19/Indonesia/JI-ITD-150Sp/2020|EPI ISL 529962|2020-03-16
hCoV-19/Indonesia/JI-ITD-136N/2020|EPI ISL 529961|2020-03-12
hCoV-19/Indonesia/YO-UGM-200927/2020|EPI ISL 516806|2020-05-16
hCoV-19/Indonesia/SA-EIJK-06/2020|EPI ISL 467375|2020-03-23
hCoV-19/Indonesia/JI-ITD-3101NT/2020|EPI ISL 458083|2020-04-11
hCoV-19/Indonesia/JK-EIJK-03/2020|EPI ISL 437191|2020-03-27
hCoV-19/Indonesia/JK-EIJK-02/2020|EPI ISL 437190|2020-03-26
hCoV-19/Indonesia/JI-ITD-853Sp/2020|EPI ISL 437187|2020-03-25
hCoV-19/Indonesia/JK-EIJK-0317/2020|EPI ISL 435282|2020-03-19
hCoV-19/Indonesia/JK-EIJK-0141/2020|EPI ISL 435281|2020-03-17
hCoV-19/Indonesia/JK-EIJK45/2020|EPI ISL 574614|2020-06-04
hCoV-19/Indonesia/EJ-ITD-3601NT/2020|EPI ISL 560991|2020-04-14
hCoV-19/Indonesia/JI-ITD-1238Sp/2020|EPI ISL 458079|2020-03-30
C
C
CoV-19/Indonesia/JI-ITD-150Sp/2020|EPI ISL 529962|2020-03-16
V-19/Indonesia/JI-ITD-136N/2020|EPI ISL 529961|2020-03-12
CoV-19/Indonesia/YO-UGM-200927/2020|EPI ISL 516806|2020-05-16
hCoV-19/Indonesia/SA-EIJK-06/2020|EPI ISL 467375|2020-03-23
hCoV-19/Indonesia/JI-ITD-3101NT/2020|EPI ISL 458083|2020-04-11
hCoV-19/Indonesia/JK-EIJK-03/2020|EPI ISL 437191|2020-03-27
hCoV-19/Indonesia/JK-EIJK-02/2020|EPI ISL 437190|2020-03-26
hCoV-19/Indonesia/JI-ITD-853Sp/2020|EPI ISL 437187|2020-03-25
hCoV-19/Indonesia/JK-EIJK-0317/2020|EPI ISL 435282|2020-03-19
hCoV-19/Indonesia/JK-EIJK-0141/2020|EPI ISL 435281|2020-03-17
hCoV-19/Indonesia/JK-EIJK45/2020|EPI ISL 574614|2020-06-04
hCoV-19/Indonesia/EJ-ITD-3601NT/2020|EPI ISL 560991|2020-04-14
hCoV-19/Indonesia/JI-ITD-1238Sp/2020|EPI ISL 458079|2020-03-30
hCoV-19/Indonesia/JK-EIJK37/2020|EPI ISL 766046|2020-05-10
hCoV-19/Indonesia/JK-EIJK28/2020|EPI ISL 766040|2020-04-17
hCoV-19/Indonesia/JK-EIJK44/2020|EPI ISL 766047|2020-06-04
hCoV-19/Indonesia/JK-EIJK35/2020|EPI ISL 766045|2020-04-27
hCoV-19/Indonesia/BT-EIJK30/2020|EPI ISL 766042|2020-04-18
hCoV-19/Indonesia/NT-EIJK27/2020|EPI ISL 766039|2020-04-14
hCoV-19/Indonesia/JK-EIJK25/2020|EPI ISL 766038|2020-04-13
hCoV-19/Indonesia/BT-EIJK17/2020|EPI ISL 766034|2020-04-09
hCoV-19/Indonesia/YO-EIJK08/2020|EPI ISL 766032|2020-03-16
hCoV-19/Indonesia/JI-ITD-1273NT/2020|EPI ISL 458081|2020-03-30
hCoV-19/Indonesia/JI-ITD-1273V/2020|EPI ISL 529964|2020-03-30
hCoV-19/Indonesia/JK-EIJK47/2020|EPI ISL 574616|2020-06-08
hCoV-19/Indonesia/JK-EIJK-2444/2020|EPI ISL 435283|2020-03-30
hCoV-19/Indonesia/JK-EIJK31/2020|EPI ISL 766043|2020-04-22
hCoV-19/Indonesia/JK-EIJK-01/2020|EPI ISL 437189|2020-03-26
hCoV-19/Indonesia/KI-EIJK-05/2020|EPI ISL 467374|2020-03-18
hCoV-19/Indonesia/JK-EIJK-39/2020|EPI ISL 568690|2020-05-26
hCoV-19/Indonesia/JK-EIJK33/2020|EPI ISL 766044|2020-04-23
hCoV-19/Indonesia/JK-EIJK-07/2020|EPI ISL 467376|2020-04-24
hCoV-19/Indonesia/JB-EIJK16/2020|EPI ISL 574606|2020-04-06
hCoV-19/Indonesia/JK-NIHRD-C0023408/2020|EPI ISL 833130|2020-06-26
hCoV-19/Indonesia/NT-EIJK14/2020|EPI ISL 766033|2020-03-28
hCoV-19/Indonesia/BGR-LIPI011/2020|EPI ISL 833494|2020-07-06
hCoV-19/Indonesia/JB-EIJK18/2020|EPI ISL 766035|2020-04-08
hCoV-19/Indonesia/JK-EIJK22/2020|EPI ISL 766037|2020-04-13
hCoV-19/Indonesia/JK-EIJK29/2020|EPI ISL 766041|2020-04-17
hCoV-19/Indonesia/JK-EIJK-04/2020|EPI ISL 437192|2020-04-01
hCoV-19/Indonesia/JI-ITDua-1006NTv/2020|EPI ISL 759961|2020-03-27
hCoV-19/Indonesia/NT-EIJK26/2020|EPI ISL 766048|2020-04-14
hCoV-19/Indonesia/JI-ITDua-998NTv/2020|EPI ISL 759959|2020-03-30
NC 045512.2:21563-25384 Severe acute respiratory syndrome coronavirus 2 isolate Wuhan-Hu-1 complete genome
0.00050

(a)

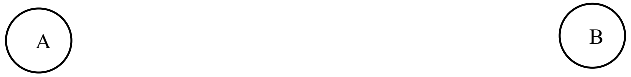
A
B

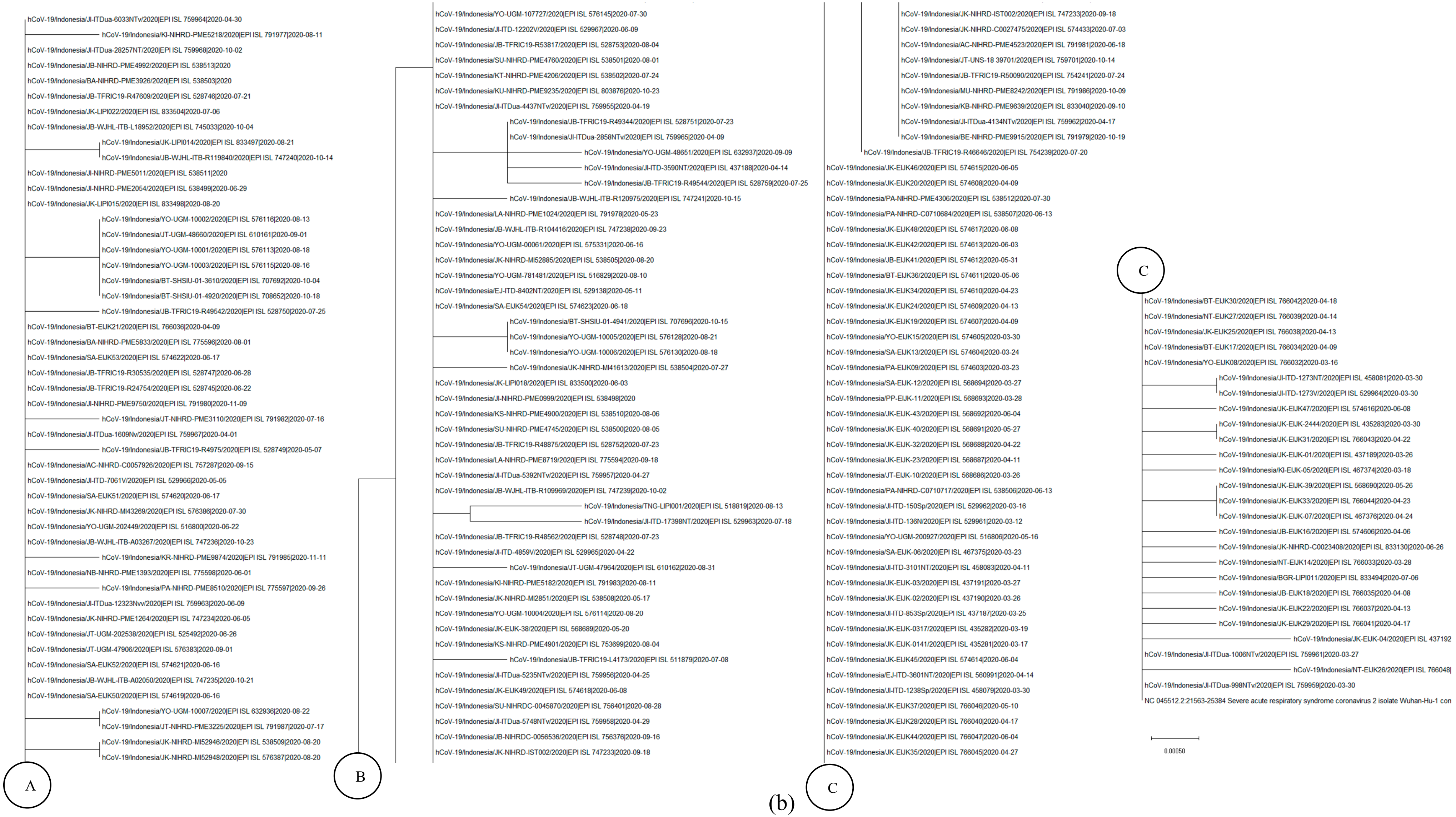

Figure S1. Phylogenetic Tree Results from: (a) Neighbor-Joining Method (b) Maximum Likelihood Method

Table S1. Wuhan-Hu-1 and EIJK-61453 epitopes population coverage

Epitope	Coverage Class I and II	HLA allele (genotypic frequency (%)) HLA-A*01:01 -2.57	HLA-A*02:03 -3.94	HLA-A*26:01 -1.21	HLA-A*32:01 -0.42	HLA-B*07:02 -0.87	HLA-B*15:02 -11.91	HLA-B*15:17 -0.9	HLA-B*40:01 -3.65	HLA-B*44:03 -9.1	HLA-B*51:01 -3.22	HLA-B*58:01 -5.81	HLA-DPA1*01:03 0	HLA-DPB1*04:01 -14.91	HLA-DRB1*04:02 -0.57	HLA-DRB1*04:03 -1.1	HLA-DRB1*04:05 -1.8	HLA-DRB1*14:01 -0.82	HLA-DRB1*15:01 -4.53	Total HLA hits
Epitope #1: SPRRARSVA	1.71%	-	-	-	-	+	-	-	-	-	-	-	-	-	-	-	-	-	-	1
Epitope #2: KIYSKHTPI	8.48%	-	+	-	+	-	-	-	-	-	-	-	-	-	-	-	-	-	-	2
Epitope #3: AEIRASANL	23.53%	-	-	-	-	-	-	-	+	+	-	-	-	-	-	-	-	-	-	2
Epitope #4: QLTPTWRVY	22.07%	-	-	-	-	-	+	-	-	-	-	-	-	-	-	-	-	-	-	1
Epitope #5: WTAGAAAYY	9.01%	+	-	+	-	-	-	+	-	-	-	-	-	-	-	-	-	-	-	3
Epitope #6: IAIPTNFTI	18.59%	-	-	-	-	-	-	+	-	-	+	+	-	-	-	-	-	-	-	3
Epitope #7: GINITRFQTLLALHR	11.46%	-	-	-	-	-	-	-	-	-	-	-	-	-	+	+	-	-	+	3
Epitope #8: HWFVTQRNFYEPQII	3.40%	-	-	-	-	-	-	-	-	-	-	-	-	-	-	-	+	-	-	1
Epitope #9: CTFEYVSQPFLMDLE	27.60%	-	-	-	-	-	-	-	-	-	-	-	+	+	-	-	-	-	-	2
Epitope #10: RFQTLLALHRSYLTP	1.56%	-	-	-	-	-	-	-	-	-	-	-	-	-	-	-	-	+	-	1
**Epitope set**	**78.26%**	**1**	**1**	**1**	**1**	**1**	**1**	**2**	**1**	**1**	**1**	**1**	**1**	**1**	**1**	**1**	**1**	**1**	**1**	**19**

+ : restricted
- : not restricted
shaded column : genotypic frequency of this allele is 0 (zero)

Table S2. UGM0002 epitopes population coverage

Epitope	Coverage Class I and II	HLA allele (genotypic frequency (%))																				Total HLA hits
		HLA-A*01:01 -2.57	HLA-A*02:03 -3.94	HLA-A*03:01 -2.37	HLA-A*11:01 -16.21	HLA-A*26:01 -1.21	HLA-A*30:01 -1.06	HLA-A*32:01 -0.42	HLA-B*07:02 -0.87	HLA-B*15:02 -11.91	HLA-B*15:17 -0.9	HLA-B*40:01 -3.65	HLA-B*44:03 -9.1	HLA-B*51:01 -3.22	HLA-B*58:01 -5.81	HLA-DPA1*01:03 0	HLA-DPB1*04:01 -14.91	HLA-DRB1*04:02 -0.57	HLA-DRB1*04:03 -1.1	HLA-DRB1*04:05 -1.8	HLA-DRB1*15:01 -4.53	
Epitope #1: SPRRARSVA	1.71%	-	-	-	-	-	-	-	+	-	-	-	-	-	-	-	-	-	-	-	-	1
Epitope #2: KIYSKHTPI	8.48%	-	+	-	-	-	-	+	-	-	-	-	-	-	-	-	-	-	-	-	-	2
Epitope #3: AEIRASANL	23.53%	-	-	-	-	-	-	-	-	-	-	+	+	-	-	-	-	-	-	-	-	2
Epitope #4: GVYFASTEK	35.25%	-	-	+	+	-	+	-	-	-	-	-	-	-	-	-	-	-	-	-	-	3
Epitope #5: QLTPTWRVY	22.07%	-	-	-	-	-	-	-	-	+	-	-	-	-	-	-	-	-	-	-	-	1
Epitope #6: WTAGAAAYY	9.01%	+	-	-	-	+	-	-	-	-	+	-	-	-	-	-	-	-	-	-	-	3
Epitope #7: IAIPTNFTI	18.59%	-	-	-	-	-	-	-	-	-	+	-	-	+	+	-	-	-	-	-	-	3
Epitope #8: GINITRFQTLLALHR	11.46%	-	-	-	-	-	-	-	-	-	-	-	-	-	-	-	-	+	+	-	+	3
Epitope #9: HWFVTQRNFYEPQII	3.40%	-	-	-	-	-	-	-	-	-	-	-	-	-	-	-	-	-	-	+	-	1
Epitope #10: CTFEYVSQPFLMDLE	27.60%	-	-	-	-	-	-	-	-	-	-	-	-	-	-	+	+	-	-	-	-	2
Epitope #11: NCTFEYVSQPFLMDL	27.60%	-	-	-	-	-	-	-	-	-	-	-	-	-	-	+	+	-	-	-	-	2
Epitope set	86.29%	1	1	1	1	1	1	1	1	1	2	1	1	1	1	2	2	1	1	1	1	23

+ : restricted

- : not restricted

shaded column : genotypic frequency of this allele is 0 (zero)

Table S3. B.1.1.7 epitopes population coverage

Epitope	Coverage	HLA allele (genotypic frequency (%))														Total HLA hits
	Class I and II	HLA-A*01:01	HLA-A*02:03	HLA-A*03:01	HLA-A*11:01	HLA-A*26:01	HLA-A*30:01	HLA-A*32:01	HLA-B*15:02	HLA-B*15:17	HLA-B*40:01	HLA-B*44:03	HLA-DPA1*02:01	HLA-DPB1*05:01	HLA-DRB1*04:02	
		-2.57	-3.94	-2.37	-16.21	-1.21	-1.06	-0.42	-11.91	-0.9	-3.65	-9.1	0	-26.39	-0.57	
Epitope #1: KIYSKHTPI	8.48%	-	+	-	-	-	-	+	-	-	-	-	-	-	-	2
Epitope #2: AEIRASANL	23.53%	-	-	-	-	-	-	-	-	-	+	+	-	-	-	2
Epitope #3: WTAGAAAYY	9.01%	+	-	-	-	+	-	-	-	+	-	-	-	-	-	3
Epitope #4: GVYFASTEK	35.25%	-	-	+	+	-	+	-	-	-	-	-	-	-	-	3
Epitope #5: QLTPTWRVY	22.07%	-	-	-	-	-	-	-	+	-	-	-	-	-	-	1
Epitope #6: RAAEIRASANLAATK	1.08%	-	-	-	-	-	-	-	-	-	-	-	-	-	+	1
Epitope #7: KHTPINLVRDLPQGF	1.08%	-	-	-	-	-	-	-	-	-	-	-	-	-	+	1
Epitope #8: KGIYQTSNFRVQPTE	45.82%	-	-	-	-	-	-	-	-	-	-	-	+	+	-	2
Epitope set	84.28%	1	1	1	1	1	1	1	1	1	1	1	1	1	2	15

+ : restricted

- : not restricted

shaded column: genotypic frequency of this allele is 0 (zero)

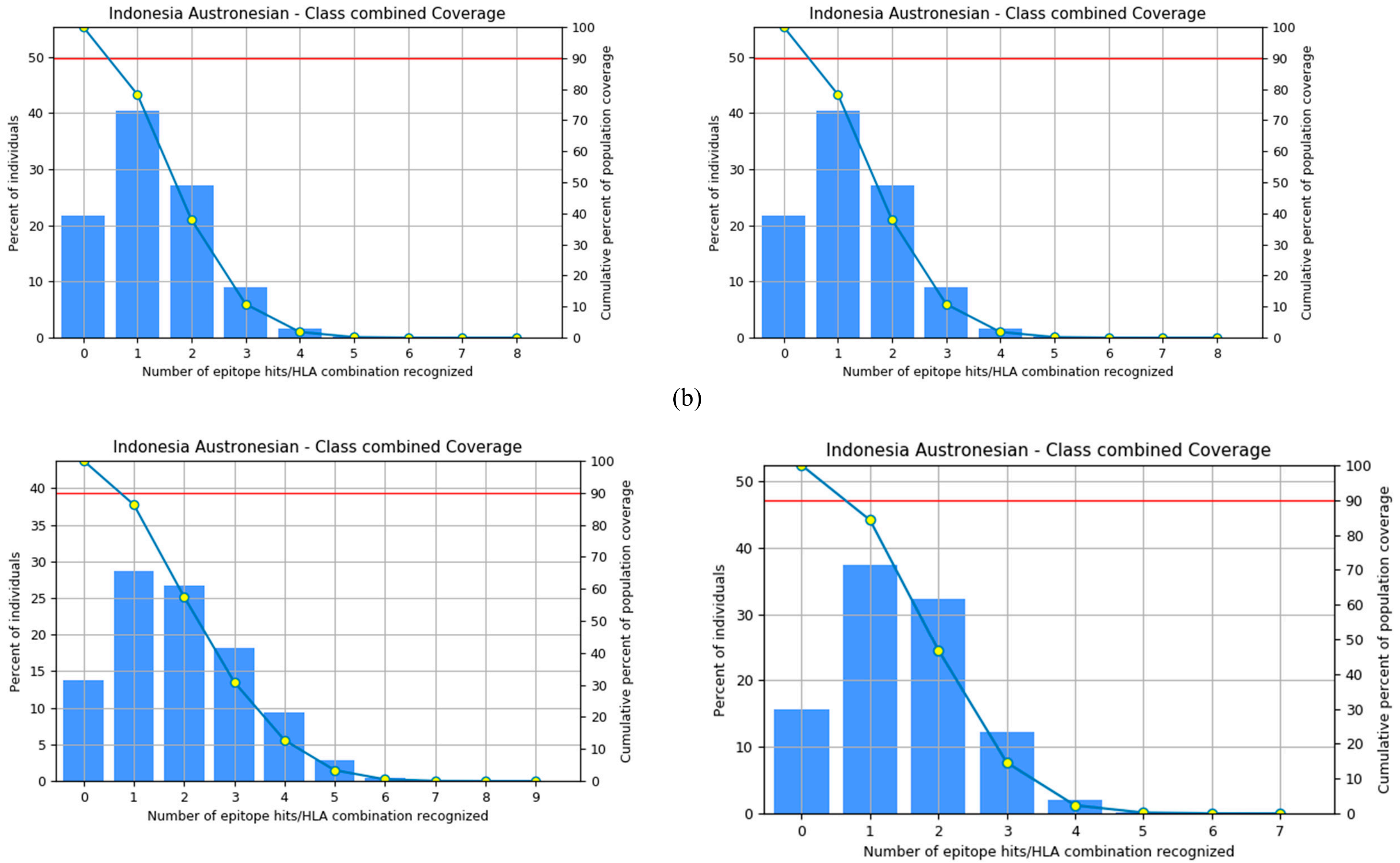

(a) (b) (c) (d)

Figure S2. Number of epitopes recognized by HLA from Indonesia population for Wuhan-Hu-1 (a), EIJK-61453 (b), UGM0002 (c), and B.1.1.7 (d)

Table S4. The predicted discontinuous B-cell epitopes

Epitope	Epitope position
	**Wuhan-Hu-1**
GSVASQSI	C: G685; C: S686; C: V687; C: A688; C: S689; C: Q690; C: S691; C: I692
FHVNNSYAY	A: F643; A: H655; A: V656; A: N657; A: N658. A: S659; A: Y660; A: A694; A: Y695
	**EIJK-61453**
GSVASQSI	C: G685; C: S686; C: V687; C: A688; C: S689. C: Q690; C: S691; C: I692
FHVNNSYAY	A: F643; A: H655; A: V656; A: N657; A: N658. A: S659; A: Y660; A: A694; A: Y695
	**UGM0002**
IRSTGSNVGAEHVNNSY	B: I624; B: R634; B: S637; B: T638; B: G639; B: S640; B: N641; B: V642; B: G652; B: A653; B: E654; B: H655; B: V656. B: N657; B: N658; B: S659; B: Y660
HVNNSY	A: H655; A: V656; A: N657; A: N658; A: S659; A: Y660
	**B.1.1.7**
IRSTGSNVGAEHVNNSY	B: I624; B: R634; B: S637. B: T638; B: G639; B: S640; B: N641; B: V642. B: G652; B: A653; B: E654; B: H655; B: V656; B: N657; B: N658; B: S659; B: Y660
HVNNSY	A: H655; A: V656; A: N657; A: N658; A: S659; A: Y660

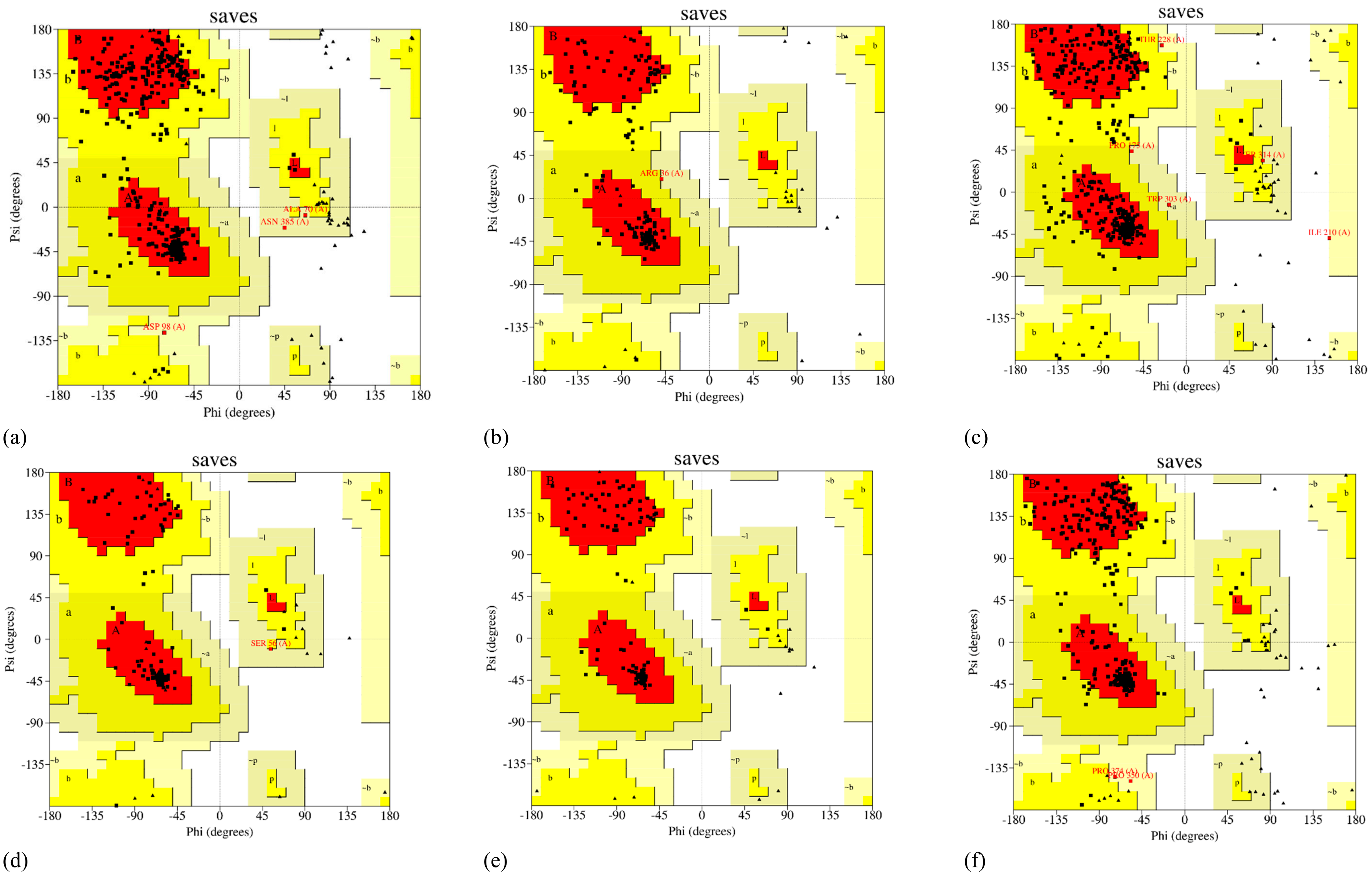

Figure S3. Ramachandran plot for (a) Wuhan-Hu-1-2 and EIJK-61453-2 with 454 aa, 88.2% in the most-favorable region, 59 Glycine residues, and 38 Proline residues.

(b) Wuhan-Hu-1-3 and EIJK-61453-3 with 189 AA, 86.7% in the most favorable region, 26 Glycine residues, and 18 Proline residues

(c) UGM0002-2 with 493 AA, 84.8% in the most favorable region, 17 Glycine residues, and 9 Proline residues

(d) UGM0002-3 with 454 AA, 88.2% in the most-favorable region, 59 Glycine residues, and 38 Proline residues

(e) B.1.1.7-1 with 140 AA, 93.8% in the most favorable region, 17 Glycine residues, and 9 Proline residues

(f) B.1.1.7-1-3 with 395 AA, 86.4% in the most favorable region, 49 Glycine residues, and 35 Proline residues

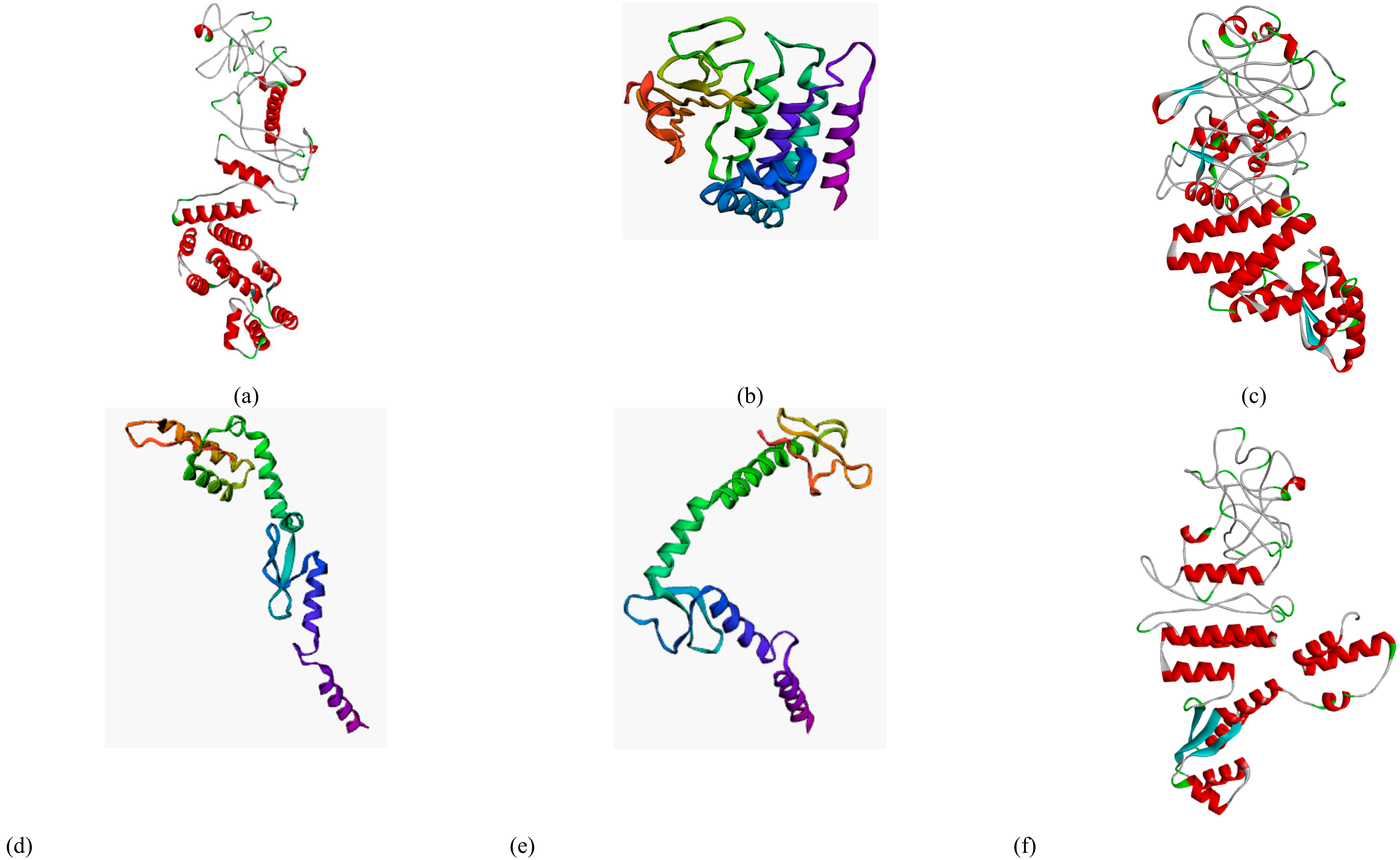

Figure S4. 3D structure prediction of (a) Wuhan-Hu-1-2 and EIJK-61453-2 vaccine, (b) Wuhan-Hu-1-3 and EIJK-61453-3 vaccine (c) UGM0002-2, (d) UGM0002-3, (e) B.1.1.7-1-1, and (e) B.1.1.7-1-3

Table S5. Top three list of vaccine construction sequences

Model	Vaccine Sequence	Score	Explanation
**Wuhan-Hu-1**			
1	MRIHYLLFALLFLFLVPVPGHGGIINTLQKYYCRVRGGRCAVLSCLPKEEQIGKCSTRGRKCCRRKK**EAAAK**SPRRARSVA**KK**WTAGAAAYY**GPGPG**GINITRFQTLLALHR**GPGPG**CTFEYVSQPFLMDLE**GPGPG**GQSKRVDFC	Quality Factor<br>ERRAT = 91,73<br>TM-Score = 0,383<br>*Template* = 1KJ6_A<br>Confidence = 97,0%	Using the template protein 1KJ6_A with 97.0% confidence<br>Vaccine length 146 aa
2	MAKLSTDELLDAFKEMTLLELSDFVKKFEETFEVTAAAPVAVAAAGAAPAGAAVEAAEEQSEFDVILEAAGDKKIGVIKVVREIVSGLGLKEAKDLVDGAPKPLLEKVAKEAADEAKAKLEAAGATVTVK**EAAAK**KIYSKHTPI**AAY**WTAGAAAYY**AAY**QLTPTWRVY**AAY**SPRRARSVA**AAY**AEIRASANL**GPGPG**CTFEYVSQPFLMDLE**GPGPG**GINITRFQTLLALHR**GPGPG**IIAYTMSLGAENSV**GPGPG**HWFVTQRNFYEPQII**GPGPG**FSNVTWFHAIHVSGTNGTKRFDN**GPGPG**NITNLCPFGEVFNATRFASVYAWNRK**GPGPG**NSASFSTFKCYGVSPTKLNDLCFTNV**GPGPG**GDEVRQIAPGQTGKIADYNYK**GPGPG**NNLDSKVGGNYNY**GPGPG**FQPTNG**GPGPG**GQSKRVDFC**GPGPG**SCCKFDEDDSEPVLKGVKL	Quality Factor<br>ERRAT = 83,18<br>TM-Score = 0,148<br>*Template* = 6YLA<br>Confidence = 100%	Using the 6YLA protein template with 100% confidence<br>Vaccine length 454 aa
3	MRIHYLLFALLFLFLVPVPGHGGIINTLQKYYCRVRGGRCAVLSCLPKEEQIGKCSTRGRKCCRRKK**EAAAK**SPRRARSVA**AAY**KIYSKHTPI**GPGPG**GINITRFQTLLALHR**GPGPG**CTFEYVSQPFLMDLE**GPGPG**FQPTNG**GPGPG**AYTMSLGAENSVAYSN**GPGPG**SCCKFDEDDSEPVLKGVKL	Quality Factor<br>ERRAT = 85,48<br>TM-Score = 0,197	Using a de novo folding approach<br>Vaccine length 189 aa
**EIJK-61453**			
1	MRIHYLLFALLFLFLVPVPGHGGIINTLQKYYCRVRGGRCAVLSCLPKEEQIGKCSTRGRKCCRRKK**EAAAK**SPRRARSVA**KK**WTAGAAAYY**GPGPG**GINITRFQTLLALHR**GPGPG**CTFEYVSQPFLMDLE**GPGPG**GQSKRVDFC	Quality Factor<br>ERRAT = 91,73<br>TM-Score = 0,383<br>*Template* = 1KJ6_A<br>Confidence = 97,0%	Using the template protein 1KJ6_A with 97.0% confidence<br>Vaccine length 146 aa
2	MAKLSTDELLDAFKEMTLLELSDFVKKFEETFEVTAAAPVAVAAAGAAPAGAAVEAAEEQSEFDVILEAAGDKKIGVIKVVREIVSGLGLKEAKDLVDGAPKPLLEKVAKEAADEAKAKLEAAGATVTVK**EAAAK**KIYSKHTPI**AAY**WTAGAAAYY**AAY**QLTPTWRVY**AAY**SPRRARSVA**AAY**AEIRASANL**GPGPG**CTFEYVSQPFLMDLE**GPGPG**GINITRFQTLLALHR**GPGPG**IIAYTMSLGAENSV**GPGPG**HWFVTQRNFYEPQII**GPGPG**FSNVTWFHAIHVSGTNGTKRFDN**GPGPG**NITNLCPFGEVFNATRFASVYAWNRK**GPGPG**NSASFSTFKCYGVSPTKLNDLCFTNV**GPGPG**GDEVRQIAPGQTGKIADYNYK**GPGPG**NNLDSKVGGNYNY**GPGPG**FQPTNG**GPGPG**GQSKRVDFC**GPGPG**SCCKFDEDDSEPVLKGVKL	Quality Factor<br>ERRAT = 83,16<br>TM-Score = 0,148<br>*Template* = 6YLA<br>Confidence = 100%	Using the 6YLA protein template with 100% confidence<br>Vaccine length 454 aa
3	MRIHYLLFALLFLFLVPVPGHGGIINTLQKYYCRVRGGRCAVLSCLPKEEQIGKCSTRGRKCCRRKK**EAAAK**SPRRARSVA**AAY**KIYSKHTPI**GPGPG**GINITRFQTLLALHR**GPGPG**CTFEYVSQPFLMDLE**GPGPG**FQPTNG**GPGPG**AYTMSLGAENSVAYSN**GPGPG**SCCKFDEDDSEPVLKGVKL	Quality Factor<br>ERRAT = 85,48<br>TM-Score = 0,197	Using a de novo folding approach<br>Vaccine length 189 aa
**UGM0002**			
1	MRIHYLLFALLFLFLVPVPGHGGIINTLQKYYCRVRGGRCAVLSCLPKEEQIGKCSTRGRKCCRRKK**EAAAK**SPRRARSVA**AAY**KIYSKHTPI**AAY**GVYFASTEK**AAY**WTAGAAAYY**GPGPG**GINIFQTLLALHRTR**GPGPG**GQSKRVDFC	Quality Factor<br>ERRAT = 99,27<br>TM-Score = 0,358	Using a de novo folding approach<br>Vaccine length 151 aa

2	MAKLSTDELLDAFKEMTLLELSDFVKKFEETFEVTAAAPVAVAAAGAAPAGAAVEAAEEQSEFDVILEAAGDKKIGVIKVVREIVSGLGLKEAKDLVDGAPKPLLEKVAKEAADEAKAKLEAAGATVTVK**EAAAK**GVYFASTEK**AAY**KIYSKHTPI**AAY**WTAGAAAYY**AAY**QLTPTWRVY**AAY**SPRRARSVA**AAY**IAIPTNFTI**AAY**AEIRASANL**GPGPG**SQCVNLTTRTQLPPAYTNSFTRGVY**GPGPG**FSNVTWFHAIHVSGTNGTKRFDNP**GPGPG**NITNLCPFGEVFNATRFASVYAWNRK**GPGPG**SASFSTFKCYGVSPTKLNDLCFTNV**GPGPG**VIRGDEVRQIAPGQTGKIADYNYKL**GPGPG**NNLDSKVGGNYNY**GPGPG**SNKKFLPF**GPGPG**TNTSNQ**GPGPG**VNCTEV**GPGPG**GQSKRVDFC**GPGPG**CTFEYVSQPFLMDLE**GPGPG**GINITRFQTLLALHR**GPGPG**HWFVTQRNFYEPQII	Quality Factor<br>ERRAT = 75,74<br>TM Score = 0,150<br>*Template* = 6XMO<br>*Confidence* = 100%	Using the 6XMO protein template with 100% confidence<br>Vaccine length 493 aa
3	MRIHYLLFALLFLFLVPVPGHGGIINTLQKYYCRVRGGRCAVLSCLPKEEQIGKCSTRGRKCCRRKK**EAAAK**SPRRARSVA**KK**KIYSKHTPI**KK**GVYFASTEK**KK**WTAGAAAYY**GPGPG**GINIFQTLLALHRTR**GPGPG**GQSKRVDFC	Quality Factor<br>ERRAT = 94,93<br>TM Score = 0,358	Using a de novo folding approach<br>Vaccine length 148 aa
**B.1.1.7**			
1	MRIHYLLFALLFLFLVPVPGHGGIINTLQKYYCRVRGGRCAVLSCLPKEEQIGKCSTRGRKCCRRKK**EAAAK**KIYSKHTPI**AAY**WTAGAAAYY**AAY**GVYFASTEK**GPGPG**KGIYQTSNFRVQPTE**GPGPG**LGQSKRVDFC	Quality Factor<br>ERRAT = 94,21<br>TM-Score = 0,361<br>*Template* = 2LWL_A<br>Confidence = 97,7%	Using the 2LWL_A protein template with 97.7% confidence<br>Vaccine length 140 aa
2	MRIHYLLFALLFLFLVPVPGHGGIINTLQKYYCRVRGGRCAVLSCLPKEEQIGKCSTRGRKCCRRKK**EAAAK**KIYSKHTPI**AAY**WTAGAAAYY**GPGPG**RAAEIRASANLAATK**GPGPG**LGQSKRVDFC	Quality Factor<br>ERRAT = 97,32<br>TM-Score = 0,405<br>*Template* = 6VSJ_C<br>Confidence = 100%	Using the 6VSJ_C protein template with 100% confidence<br>Vaccine length 128 aa
3	MAKLSTDELLDAFKEMTLLELSDFVKKFEETFEVTAAAPVAVAAAGAAPAGAAVEAAEEQSEFDVILEAAGDKKIGVIKVVREIVSGLGLKEAKDLVDGAPKPLLEKVAKEAADEAKAKLEAAGATVTVK**EAAAK**GVYFASTEK**AAY**KIYSKHTPI**AAY**WTAGAAAYY**AAY**QLTPTWRVY**AAY**AEIRASANL**GPGPG**KHTPINLVRDLPQGF**GPGPG**KGIYQTSNFRVQPTE**GPGPG**RAAEIRASANLAATK**GPGPG**SQCVNLTTRTQLPPAYTNSFTRGVY**GPGPG**YNSASFSTFKCYGVSPTKLNDLCFT**GPGPG**GDEVRQIAPGQTGKIA**GPGPG**SNKKFLPF**GPGPG**VNCTEV**GPGPG**LGQSKRVDFC**GPGPG**SCCKFDEDDSEPVLKGVK	Quality Factor<br>ERRAT = 87,89<br>TM-Score = 0,22	Using a de novo folding approach<br>Vaccine length 395 aas

Table S6. Optimized vaccine sequences

EIJK
Pasted Sequence: GC=68.72%, CAI=0.58
ATGAGGATCCACTACCTGCTGTTCGCCCTGCTGTTCCTGTTCCTGGTGCCCGTGCCCGGCCACGGCGGCATCATCAACACCCTGCAGAAGTACTAC<br>TGCAGGGTGAGGGGCGGCAGGTGCGCCGTGCTGAGCTGCCTGCCCAAGGAGGAGCAGATCGGCAAGTGCAGCACCAGGGGCAGGAAGTGCTGCA<br>GGAGGAAGAAGGAGGCCGCCGCCAAGAGCCCCAGGAGGGCCAGGAGCGTGGCCAAGAAGTGGACCGCCGGCGCCGCCGCCTACTACGGCCCCG<br>GCCCCGGCGGCATCAACATCACCAGGTTCCAGACCCTGCTGGCCCTGCACAGGGGCCCCGGCCCCGGCTGCACCTTCGAGTACGTGAGCCAGCCC<br>TTCCTGATGGACCTGGAGGGCCCCGGCCCCGGCGGCCAGAGCAAGAGGGTGGACTTCTGC
Improved DNA[1]: GC=59.82%, CAI=0.93
ATGCGTATTCACTACCTGCTGTTTGCCCTGCTGTTTCTGTTTCTGGTGCCGGTGCCGGGCCATGGCGGCATTATTAACACCCTGCAGAAATATTATT<br>GCCGCGTGCGCGGTGGCCGCTGCGCCGTTCTGAGCTGTCTGCCGAAAGAAGAACAGATCGGTAAATGTAGCACCCGCGGCCGTAAATGCTGCCGT<br>CGTAAAAAAGAAGCGGCGGCGAAATCACCGCGCCGTGCCCGTAGCGTGGCAAAAAAATGGACCGCCGGCGCCGCGGCGTACTATGGCCCGGGTC<br>CGGGCGGCATTAACATTACCCGCTTTCAGACCCTGCTGGCCCTGCATCGCGGTCCGGGCCCGGGCTGCACCTTTGAATATGTGTCGCAGCCGTTTC<br>TGATGGATCTGGAAGGCCCGGGCCCGGGCGGCCAAAGCAAACGCGTTGATTTTTGT
UGM
Pasted Sequence: GC=68.21%, CAI=0.59
ATGAGGATCCACTACCTGCTGTTCGCCCTGCTGTTCCTGTTCCTGGTGCCCGTGCCCGGCCACGGCGGCATCATCAACACCCTGCAGAAGTACTAC<br>TGCAGGGTGAGGGGCGGCAGGTGCGCCGTGCTGAGCTGCCTGCCCAAGGAGGAGCAGATCGGCAAGTGCAGCACCAGGGGCAGGAAGTGCTGCA<br>GGAGGAAGAAGGAGGCCGCCGCCAAGAGCCCCAGGAGGGCCAGGAGCGTGGCCGCCGCCTACAAGATCTACAGCAAGCACACCCCCATCGCCGC<br>CTACGGCGTGTACTTCGCCAGCACCGAGAAGGCCGCCTACTGGACCGCCGGCGCCGCCGCCTACTACGGCCCCGGCCCCGGCGGCATCAACATCT<br>TCCAGACCCTGCTGGCCCTGCACAGGACCAGGGGCCCCGGCCCCGGCGGCCAGAGCAAGAGGGTGGACTTCTGC
Improved DNA[1]: GC=59.82%, CAI=0.95
ATGCGCATTCATTATCTGCTGTTTGCGCTGCTGTTTCTGTTTCTGGTGCCGGTGCCGGGCCATGGCGGCATTATTAACACCCTGCAGAAATACTATT<br>GCCGCGTGCGTGGCGGCCGCTGCGCCGTGCTGAGCTGCCTGCCGAAAGAAGAACAGATTGGCAAATGCAGCACCCGCGGCCGTAAATGTTGCCGC<br>CGCAAAAAAGAAGCGGCCGCGAAAAGCCCGCGCCGCGCACGTAGCGTGGCCGCGGCATATAAAATTTACAGCAAACATACCCCGATTGCGGCCT<br>ACGGCGTGTACTTTGCGTCAACCGAAAAAGCGGCCTATTGGACCGCGGGCGCGGCGGCCTATTATGGTCCTGGCCCGGGCGGCATTAACATTTTTC<br>AGACCCTGCTGGCGCTGCATCGCACCCGCGGCCCGGGCCCGGGTGGCCAGAGTAAACGTGTGGATTTTTGC
B117

Pasted Sequence: GC=68.75%, CAI=0.59
ATGAGGATCCACTACCTGCTGTTCGCCCTGCTGTTCCTGTTCCTGGTGCCCGTGCCCGGCCACGGCGGCATCATCAACACCCTGCAGAAGTACTAC<br>TGCAGGGTGAGGGGCGGCAGGTGCGCCGTGCTGAGCTGCCTGCCCAAGGAGGAGCAGATCGGCAAGTGCAGCACCAGGGGCAGGAAGTGCTGCA<br>GGAGGAAGAAGGAGGCCGCCGCCAAGAAGATCTACAGCAAGCACACCCCCATCGCCGCCTACTGGACCGCCGGCGCCGCCGCCTACTACGGCCC<br>CGGCCCCGGCAGGGCCGCCGAGATCAGGGCCAGCGCCAACCTGGCCGCCACCAAGGGCCCCGGCCCCGGCCTGGGCCAGAGCAAGAGGGTGGAC<br>TTCTGC
Improved DNA[1]: GC=59.64%, CAI=0.95
ATGCGTATTCATTACCTGCTGTTTGCGCTGCTGTTTCTGTTTCTGGTGCCGGTGCCGGGTCATGGCGGTATTATTAACACCCTGCAGAAATATTATT<br>GTCGCGTGCGTGGCGGCCGCTGTGCGGTTCTGAGCTGCCTGCCGAAAGAAGAACAGATTGGTAAATGCAGCACCCGCGGCCGCAAATGCTGCCGT<br>CGCAAAAAAGAAGCGGCGGCGAAAAAAATTTATTCGAAACATACCCCGATTGCCGCCTATTGGACCGCGGGTGCGGCCGCGTACTATGGCCCGG<br>GCCCGGGCCGCGCGGCGGAAATCCGCGCGAGCGCAAACCTGGCGGCGACCAAAGGCCCGGGCCCGGGCCTGGGCCAGAGCAAACGCGTTGATTT<br>TTGC
